# The Functions and Applications of Fluorinated Interface Engineering in Li‐Based Secondary Batteries

**DOI:** 10.1002/smsc.202100066

**Published:** 2021-10-16

**Authors:** Mu-Yao Qi, Yan-Song Xu, Si-Jie Guo, Si-Dong Zhang, Jin-Yang Li, Yong-Gang Sun, Ke-Cheng Jiang, An-Min Cao, Li-Jun Wan

**Affiliations:** ^1^ CAS Key Laboratory of Molecular Nanostructure and Nanotechnology and Beijing National Laboratory for Molecular Sciences Institute of Chemistry Chinese Academy of Sciences (CAS) Beijing 100190 P. R. China; ^2^ School of Chemical Sciences University of Chinese Academy of Sciences Beijing 100049 P. R. China; ^3^ Dongguan TAFEL New Energy Technology Company Limited Dongguan 523000 P. R. China

**Keywords:** fluorination treatment, interphase stability of lithium metal batteries, Li-based secondary batteries, solid electrolyte interphase, surface modification of electrode materials

## Abstract

Li‐based secondary batteries are now attracting soaring research attention as a promising energy storage system with high energy density for commercial applications. However, the high‐energy systems meanwhile are causing serious concerns on safety issues due to unstable interfaces on both cathodes and anodes. To improve interphase stability upon extended cycles, surface fluorinated treatment becomes highly desirable due to its unique capability in modulating the chemistry of electrode/electrolyte interface to ensure a stable electrochemical performance. Accordingly, it is essential that a deeper understanding on the solid electrolyte interphase (SEI), especially the role of fluorine‐containing components, is demanded to guide the interface design. This review begins with an introduction to the fundamental knowledge on the structure of SEI with focus on the unique physiochemical properties of fluorides. Detailed discussions are then taken on the control strategies for a reliable construction of fluoride‐based interfaces, which typically includes the surface coating of metal fluorides on cathodes and ex situ/in situ fluorination on lithium, based on which the structure–performance relationship is elaborated to inspire a rational interface engineering. Finally, perspectives are provided to give insights into the possible research directions of fluorinated SEI for further development of rechargeable Li batteries.

## Graphical abstract

Interface modification is a general strategy to improve the interphase stability for Li‐based secondary batteries (LSBs) with high energy density. F‐containing species, particularly LiF, are usually utilized to alleviate side reactions and suppress dendrites during repeated cycling. Therefore, the functions and applications of fluorinated interfaces engineering are focused on to enable excellent performance to facilitate further development of LSBs.

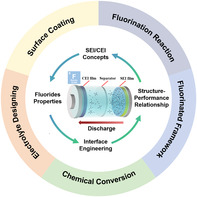

## 1 Introduction

Past few years have witnessed great progress in energy storage technology and material characterization methods, which stimulated the development of novel energy storage devices suitable for large‐scale industrial applications.^[^ ^1^, ^2^, ^3^, ^4^ ^]^ Among all the energy conversion avenues, electrochemical means have the advantages of environmental benignity, high transforming efficiency, and long lifespan, which are expected to overcome the intrinsic shortcomings of directly unavailable renewable energy sources such as wind power, tidal power, and solar energy, whose defects often lie in distribution inhomogeneity and space‐time limitations across the world.^[^ ^5^ ^]^ So, a large variety of electrochemical energy storage devices emerges as urgently demanded in reality during the past two centuries to meet different requirements in social production and residential lives. Particularly, secondary battery technology, also called rechargeable battery, is a reliable type of electrochemical system with ability to charge and discharge repeatedly taking advantage of reversible chemical reactions.^[^ ^6^, ^7^ ^]^ In contrast to primary battery first designed by Alessandro Volta in 1799, rechargeable battery systems such as nickel–metal hydride batteries,^[^ ^8^ ^]^ nickel–cadmium batteries,^[^ ^9^ ^]^ lead–acid batteries,^[^ ^10^ ^]^ lithium‐ion batteries (LIB), and polymer LIBs^[^ ^11^ ^]^ have huge potential to apply in portable electronic devices, electric vehicles (EV), implantable medical equipments, and static smart power grid due to their distinct advantages of durability, energy density, and cost performance. Accordingly, many of these have achieved great success in market during the past few decades. Specially, LIB, served as a milestone in electrochemical energy storage technology history for humankind and first commercialized by Sony in 1991 with a configuration of cobalt oxide cathodes and graphite anodes,^[^ ^12^ ^]^ has been widely deployed in 3C (Computer, Communication, Consumer electronics products) fields to revolutionize our modern life so far.

**Figure 1:**
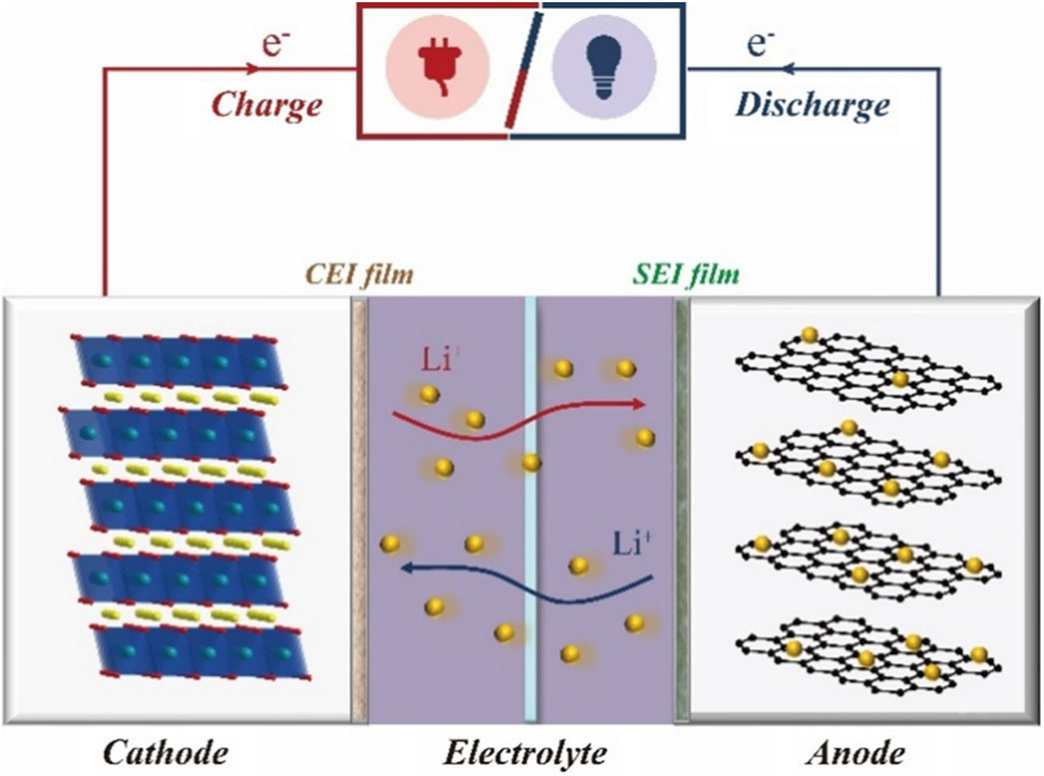
Schematic illustration of a typical LIB with lithium cobaltate (LCO) as cathode and graphite as anode materials, respectively.

As shown in **Figure** **1**, the working mechanism of LIBs usually follows a typical “rocking chair” style, which is featured by the reversible charge‐carrier (alkali metal ion) separation and recombination at the electrode/electrolyte interface in host materials including cathodes and anodes.^[^ ^7^ ^]^ To be specific, when exerted with a potential difference, a charge process drives Li^+^ to deintercalate from the lattice spacing of a cathode (layered oxides, spinel or olivine types, etc.)^[^ ^13^, ^14^ ^]^ and move toward the anode host (e.g., graphite or silicon)^[^ ^15^, ^16^ ^]^ across the electrolyte, while the electrons released by Li^+^ are guided to travel to negative side through outer wire. Finally, at a molecular level, Li^+^ ions compound with electrons and intercalate into anode lattice space. Therefore, a circuit is completed through directional movement of ions and electrons under outer voltage. For a discharge process, thoroughly opposite redox reactions happen at both positive and negative side to supply power to electrical appliances in external circuit. Although the deep insight into operation mechanism of LIBs promotes researchers to develop a series of mature material systems via synthesizing and screening suitable framework for housing Li^+^ ions, the state‐of‐the‐art battery system based on nickel‐rich ternary layered oxide cathode (LiNi_1–*x*–*y*_Co_*x*_M_*y*_O_2_, M = Mn or Al; 1–*x*–*y* ≥ 0.6) and graphite anode whose specific capacity is about 372 m Ah/g still cannot fully satisfy the increasingly practical requirements of energy capacity and calendar life.^[^ ^17^ ^]^ The upcoming target of 500 W h kg^−1^ or 500 km per charge for EV seems to be insurmountable.^[^ ^18^, ^19^ ^]^ As a result of that, for sake of high energy LIBs, lithium metal has been revisited and utilized again as the anode after a long period of meandering exploration course since 1970s when Exxon designed the first lithium secondary battery with a TiS_2_‐Li configuration (LiPF_6_ as lithium salt in dissolved in propylene carbonate [PC]).^[^ ^20^, ^21^ ^]^ However, safety concerns and poor cycling life span caused by the growth and propagation of lithium dendrites are unavoidable all along the path forward despite endowed with a ≈3860 m Ah g^−1^ relatively high specific capacity and the lowest reduction potential (−3.04 V). In addition, flammability of nonaqueous electrolyte together with incompatibility of anodic solid–liquid interphase is also a serious challenge which calls for more tremendous efforts in reasonable interface design and composition optimization.

Recently, more and more researches pointed out that the surface of electrodes is the place of side reactions and the source of material structure degradation.^[^ ^22^, ^23^ ^]^ Surface modification is not only the core treatment process of positive and negative electrode materials but also an effective method to establish functional interfacial layers.^[^ ^24^, ^25^, ^26^ ^]^ Such technology needs our basic understanding of interface evolution mechanism, ion transport chemistry, compositional structure along with recognition of the function of each component. However, this is exactly what is currently missing on account of existing knowledge and characterization tools. In fact, as early as 1979, Peled^[^ ^27^ ^]^ proposed the concept of the solid electrolyte interphase (SEI) concerning the electrochemical behavior of alkali metals in organic battery systems. He regarded the electronic insulting surface layer formed on anode side which arises from the reaction of electrolyte solution with alkali metals as SEI. After that, the voltage decay was associated with the electrolyte concentration and passivating film types to manifest a “double‐layer” model where a thin and compact inner film coexists with a secondary porous outer part.^[^ ^28^ ^]^ On the cathode side, the interface has the similar meaning as the cathode electrolyte interphase (CEI). It was systematically studied at various cathode cases in 2004 using X‐ray photoelectron spectroscopy and synchrotron radiation.^[^ ^29^ ^]^ In a word, either SEI or CEI is a highly composition‐dependent stable film formed upon initial battery operation cycles (namely, formation cycles in industry) accompanied by consumption of electrolyte and active lithium ions. Their physicochemical properties are believed to be beneficial to protect electrode surfaces from further corrosion by acidic species (e.g., HF) and mitigate continuous depletion of ingredients in electrolyte. In addition, a robust CEI has the ability to suppress the partial dissolution of transition metals (commonly used in cathodes), preventing electrolyte solution reduction at negative side against overgrowth of SEI.^[^ ^30^ ^]^ Thus, it is of paramount importance to get a comprehensive insight into interphases in lithium‐based secondary battery from fundamental understanding of composition, structure, properties, function, and evolution mechanism to practical application of electrolyte and passivation layer design. In this review, we will highlight the construction or regulation method for electrode–electrolyte interphase based on SEI theory and its application in anode side especially lithium metal that has been reckoned as the key factor to the ultimate battery performance, while relatively less space will be taken up to provide guidance for CEI design on cathode.

It is well known that the solvent, additives, and lithium salt in electrolyte have great influence in subsequent SEI formation process. No matter what kind of electrolyte or battery system is chosen as studying object, there is a competition reaction between species mentioned earlier depending on respective exchange current density and reduction potential, strongly affecting the SEI structure.^[^ ^31^ ^]^ Moreover, preferential solvation is found for lithium ions with cyclic and more polar solvent ethylene carbonate (EC) over acyclic linear carbonates such as DMC and EMC in graphite model anode.^[^ ^32^, ^33^ ^]^ Therefore, it is not inapprehensible that except for optimizing composition ratio, researchers and manufacturers use a large variety of functional film forming agents (e.g., vinylene carbonate, VC, decomposing in a potential range of 0.57–1.21 V theoretically^[^ ^34^ ^]^), F‐containing solvents (e.g., fluoroethylene carbonate, FEC), and Li salts (representative as lithium bis(fluorosulfonyl)imide, LiFSI) to conventional alkyl carbonate electrolyte, which makes SEI building process more elusive and complex. Among different types of SEI, the fluorinated one emerges as a stable anodic protective film capable of regulating Li plating/stripping behavior and reinforcing the interphase stability for lithium metal batteries (LMBs). Although the proposition that LiF plays a leading role in SEI as a main component is still controversial nowadays,^[^ ^35^, ^36^ ^]^ which has been summarized by Tan based on latest studies,^[^ ^37^ ^]^ it is no doubt that fluorides have ubiquitous effects on electronic transmission and surface diffusion of Li ions from a dendrite‐suppression point of view, proven by many applied works.^[^ ^38^ ^]^ Nevertheless, more efforts are worth being devoted to investigating fluorinated SEI because it has important enlightenment and reference significance to uncover components and nature of SEI.

Here, we will start from a brief introduction of the fundamental understanding of SEI, and then elucidate in details the physicochemical properties of fluorides, especially LiF—one of the most controversial components in spontaneous bulk SEI—to show their strengths in surface modification for LIBs cathode and artificial SEI construction for LMBs. Special attention is paid to the structure–performance relationship of fluorinated anode interphase observed in two distinctive routes that are introduction of F‐containing species via ex situ chemical reaction and electrolyte‐derived fluorinated SEI generated by in situ electrochemical reaction in view of their critical role in improved electrochemical performance. Typically, the former is universal and effective to modify the solid electrolyte or lithium metal to achieve an ionic conductive but electronically insulated (electron conductivity: ≈10^−10^ S cm^−1^) interface.^[^ ^39^ ^]^ However, the ex situ method usually requires complex operating procedures and harsh reaction conditions. It cannot contribute to homogenous SEI morphology, let alone comprehensive and precise control of composition. By contrast, F‐containing electrolyte additives and anions are simply effective to create fluorinated SEI on electrodes directly. To sum up, this review is directed to the function and application of fluorinated interface engineering, discussing from three aspects—the fundamental knowledge of SEI, the metal fluoride coating control to address cathode issues, and fluorinated SEI design for lithium metal. Finally, we will conclude and provide perspectives on challenges and strategies to raise the awareness of SEI and promote the development of next‐generation electrode materials with high energy density.

## 2 The Fundamental Understanding of SEI

As the concept of SEI was put forward, the knowledge and understanding of it has been constantly developing over the past few decades, whether from the theoretical or experimental point of view. As early as 1970, Dey discovered that a passivation film will form on the surface of lithium metal when it is soaked in organic solvent for a period of time.^[^ ^40^ ^]^ Subsequently, in 1979, Peled found that alkali metals and alkaline earth metals in nonaqueous battery systems are also covered with a surface film of at least 1.5–2.5 nm when immersed in electrolyte. It is characteristic of solid electrolyte and served as an interphase between metal electrodes and electrolyte solution. Therefore, he first introduced the term “solid electrolyte interphase (SEI).”^[^ ^27^ ^]^ To further identify the components in surface films formed on lithium, in situ X‐ray diffraction (XRD), Fourier transform infrared spectroscopy (FTIR), and X‐ray photoelectron spectroscopy (XPS) characterization techniques have been used in LiClO_4_/PC electrolyte solution by Nazri and Muller^[^ ^41^ ^]^ and Aurbach^[^ ^42^ ^]^ successively to prove the existence of lithium carbonate (Li_2_CO_3_), lithium alkyl carbonates (RCO_3_Li, R=alkyl) in 1985 and 1987. In addition, Li_2_O was observed in the presence of trace amount of water.^[^ ^41^ ^]^ These two works not only confirmed the reduced products of electrolyte solvent but also gave indication of lithium salt reduction in accordance with halide ion signals in XPS.^[^ ^42^ ^]^ Since then, various SEI components have been intensively verified and studied in liquid nonaqueous solutions, including insoluble inorganic salts (e.g., Li_2_CO_3_, LiF, LiH, Li_2_O, Li_3_N, etc.) with partial soluble degraded carbonates (alkoxides, semicarbonates, and nonconducting polymers) in the light of spectral analysis.^[^ ^43^, ^44^, ^45^, ^46^ ^]^

**Figure 2:**
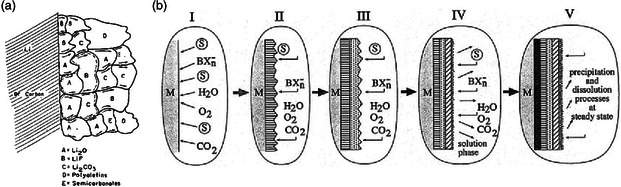
Schematic presentation of a) a mosaic‐type microphase SEI. Reproduced with permission.^[^ ^47^ ^]^ Copyright 1997, IOP Publishing. b) The formation of a multilayered SEI on active electrodes. Reproduced with permission.^[^ ^44^ ^]^ Copyright 1999, Elsevier.

**Figure 3:**
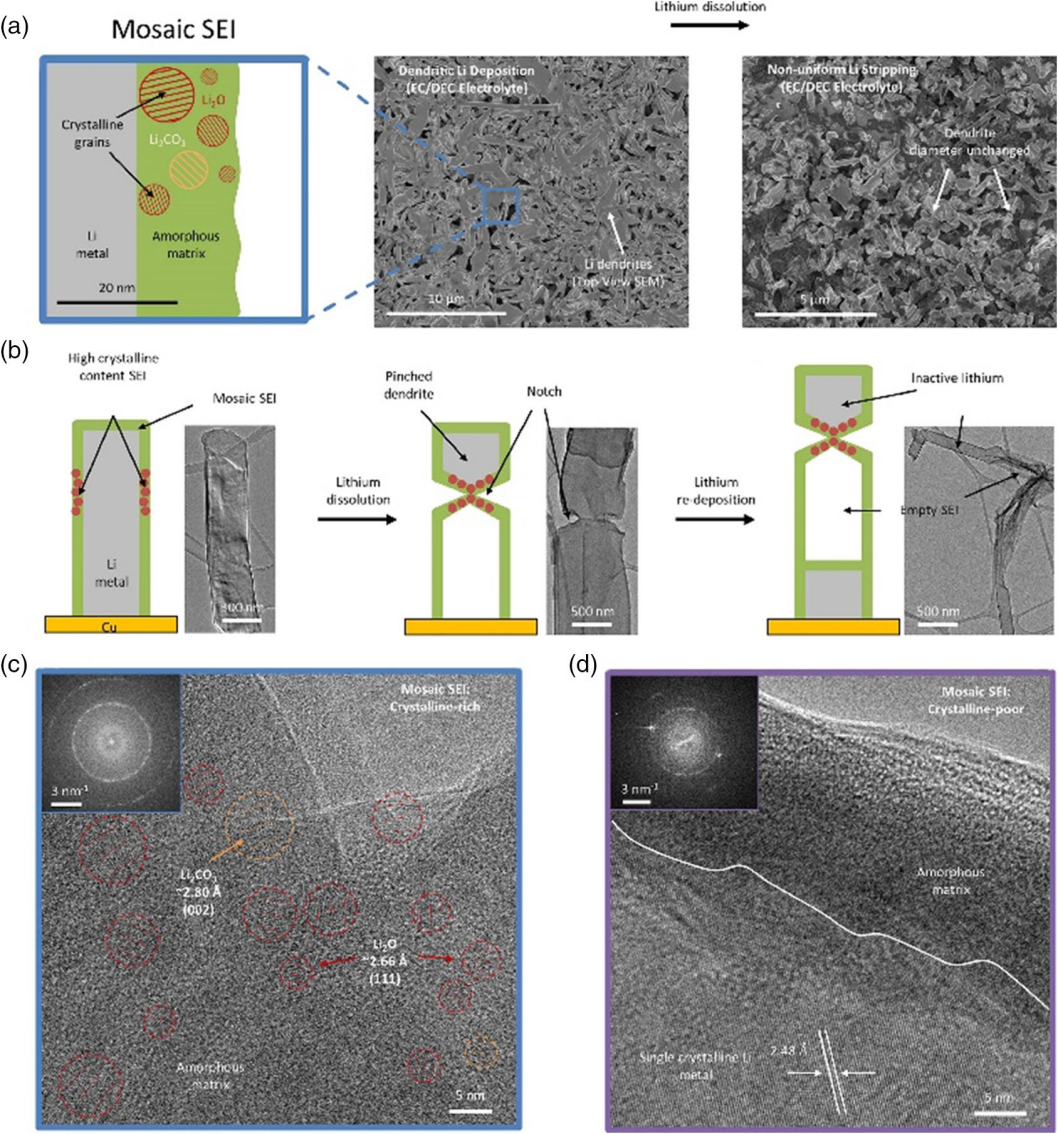
a) Schematic of the mosaic SEI nanostructure that forms on Li metal and SEM images of deposited/stripped Li metal in EC/DEC electrolyte. b) Cryo‐EM image and schematic of a typical Li metal dendrite evolution with the formation of notched structures and inactive Li metal during stripping/redeposition process. c) A high concentration of crystalline grains at the notched region of SEI nanostructure. d) Amorphous SEI away from the notch. a‐d) Reproduced with permission.^[^ ^48^ ^]^ Copyright 2018, Elsevier.

Such a complex chemical phase contribution in SEI calls for a reasonable model to interpret the morphological features (thickness, porosity, and roughness) and ion transport kinetics through the interphase. In this regard, two mainstream SEI models have been proposed by Peled^[^ ^47^ ^]^ and Aurbach^[^ ^44^ ^]^ that are mosaic model (**Figure** **2**a) and multiple layer model (Figure 2b) to describe the structure of surface film on both alkali metal and carbon electrodes. At the beginning, inspired by larger grain boundary resistance than the bulk ionic resistance in SEI, the reduced materials precipitated on anodes are considered to be dispersed randomly as separate microphases in a mosaic pattern. This type of SEI has been widely reported in recent research systems using advanced characterization techniques. For example, as shown in **Figure** **3**a, Li et al.^[^ ^48^ ^]^ observed nonuniform Li deposition/dissolution through a mosaic nanostructured SEI in conventional carbonate‐based electrolyte (1.0 m LiPF_6_ in 1:1 w w^−1^ EC/diethyl carbonate [DEC]) equipped with a Cu foil as the working electrode using cryo‐electron microscope (cryo‐EM). However, when full electrochemical discharging the deposited Li with such SEI structure to 1.0 V, only low 88% coulombic efficiency (CE) can be achieved accompanied with appearance of a large amount of inactive lithium within SEI in form of notches (Figure 3b), which is featured with loss of electrical contact with current collector. Hence, to some degree, the uneven spatial distribution of inorganic crystalline domains in SEI (Figure 3c,d) is believed to be unfavorable to suppress the formation of Li dendrites, leading to poor cyclability.

**Figure 4:**
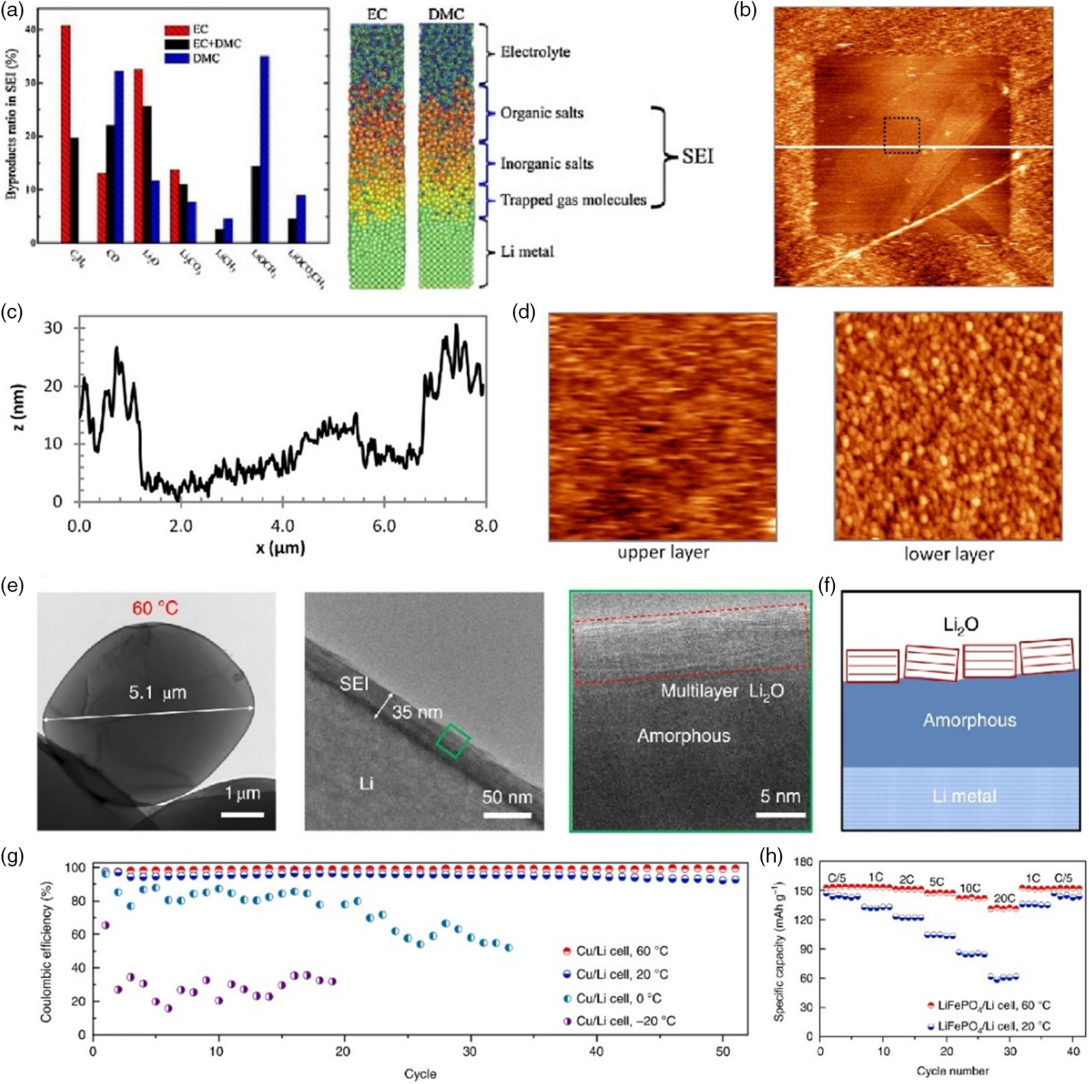
a) Distribution of the SEI components for different electrolytes (left). Atomic configurations from MD simulations (right). b) Topographic AFM image at 0 V in the control electrolyte. c) Height variation profile of SEI corresponding to the white line in (b). d) The features of the upper and lower SEI layers (black dotted square in b). e) A SEI nanostructure formed at 60 °C. Li metal was deposited in DOL/DME/LiNO_3_ using 1 m LiTFSI salt. f) Schematics of the observed thicker layered SEI structure on a Li particle grown at 60 °C. g) CE of Cu/Li cells cycled at −20, 0, 20, and 60 °C, respectively, at a current density of 1 mA cm^−2^ and a deposition capacity of 1 mAh cm^−2^. h) Rate capability of LiFePO_4_/Li cells cycled at various C‐rates (1 C = 170 mA g^−1^) with a voltage range of 2.5–3.75 V, at 20 and 60 °C. Part (a): Reproduced with permission.^[^ ^49^ ^]^ Copyright 2011, Elsevier. Parts (b‐d): Reproduced with permission.^[^ ^50^ ^]^ Copyright 2014, American Chemical Society. Parts (e‐h): Reproduced with permission.^[^ ^51^ ^]^ Copyright 2019, Springer Nature.

While under certain conditions, when Li is exposed to solution, the reduction reactions of all possible reagents first occur in a low selectivity way to block the active surface due to the high reactivity of lithium metal. Later, the electrolyte will continue to accept electrons through bottom layer and thus further reactions transform into higher selectivity. Once the concentration of surface species reaches a saturated point, a steady state will be established during dissolution and redeposition processes. Consequently, in a sense of reaction order, a multiple‐layered model may be more suitable for explaining internal and external differences in SEI from large scale. This model has also been proved by experimental and computational simulation results in previous years, and accordingly held in high esteem among battery community. For example, in the case of the mixture of EC and dimethyl carbonate (DMC), Kim et al.^[^ ^49^ ^]^ achieved a multiple‐layered surface film on lithium metal with a dense inorganic salts‐rich inner surface and a porous organic outer interface derived from EC and DMC, respectively, using a molecular dynamics (MD) simulations method (**Figure** **4**a). In addition, Cresce et al.^[^ ^50^ ^]^ took advantage of in situ atomic force microscopy (AFM) techniques not only to suggest the preference of interphasial species accumulation and solvated Li^+^ intercalation to solvent reduction in HOPG but also to characterize the structure and morphology of hierarchical SEI varying as the distance to electrode with an upper layer ranging from 10 to 480 nm in a control electrolyte (1.5 m LiTFSI/EC) (Figure 4b‐d). Furthermore, apart from carbonate‐based electrolyte, similar ordered multilayered structures can also be built up in ether‐based electrolyte through rational electrolyte design and elevated temperature. As shown in Figure 4e‐g, Wang et al.^[^ ^51^ ^]^ found that in contrast to adverse temperature effect in additive‐free carbonate electrolytes, higher temperature induces nucleation and growth of lithium oxide on amorphous SEI substrate in ether‐based electrolyte (1.0 m LiTFSI in DOL/DME/LiNO_3_) and produces an inverse layered nanostructure against continuous SEI dissolution, leading to excellent interphase stability with 99.8% CE of Cu/Li cell after 30 cycles. As a result, with the highly ordered layered SEI structure formed in ether electrolyte, the LiFePO_4_/Li half‐cell cycled at 60 °C delivered a 70 mAh g^−1^ higher reversible capacity than that at 20 °C under the same rate condition (20 C) (Figure 4h).

Although recent studies show that a multilayered‐like SEI is common in general and facilitates the regulation of interface properties, structures in reality are even more complicated involving impurities, inhomogeneous ion flux, uneven potential distribution, and side reactions.^[^ ^52^ ^]^ Different initial lithiation conditions for first few cycles, such as charging/discharging rate, potentiostatic/galvanostatic mode and times, will also significantly affect SEI quality and chemical composition, which determine the performance of the negative electrode directly.^[^ ^53^ ^]^ Intuitively, the thickness of SEI is highly depending on the sort of solvents and salts, ranging from tens of nanometers to several hundred nanometers, much higher than CEI.^[^ ^54^, ^55^, ^56^, ^57^ ^]^ Therefore, it is necessary to discuss the formation mechanism of SEI in detail so as to gain deeply insight into the structure–performance relationship for controllable interfacial design of both cathodes and anodes.

### 2.1 The Formation Mechanism of SEI

**Figure 5:**
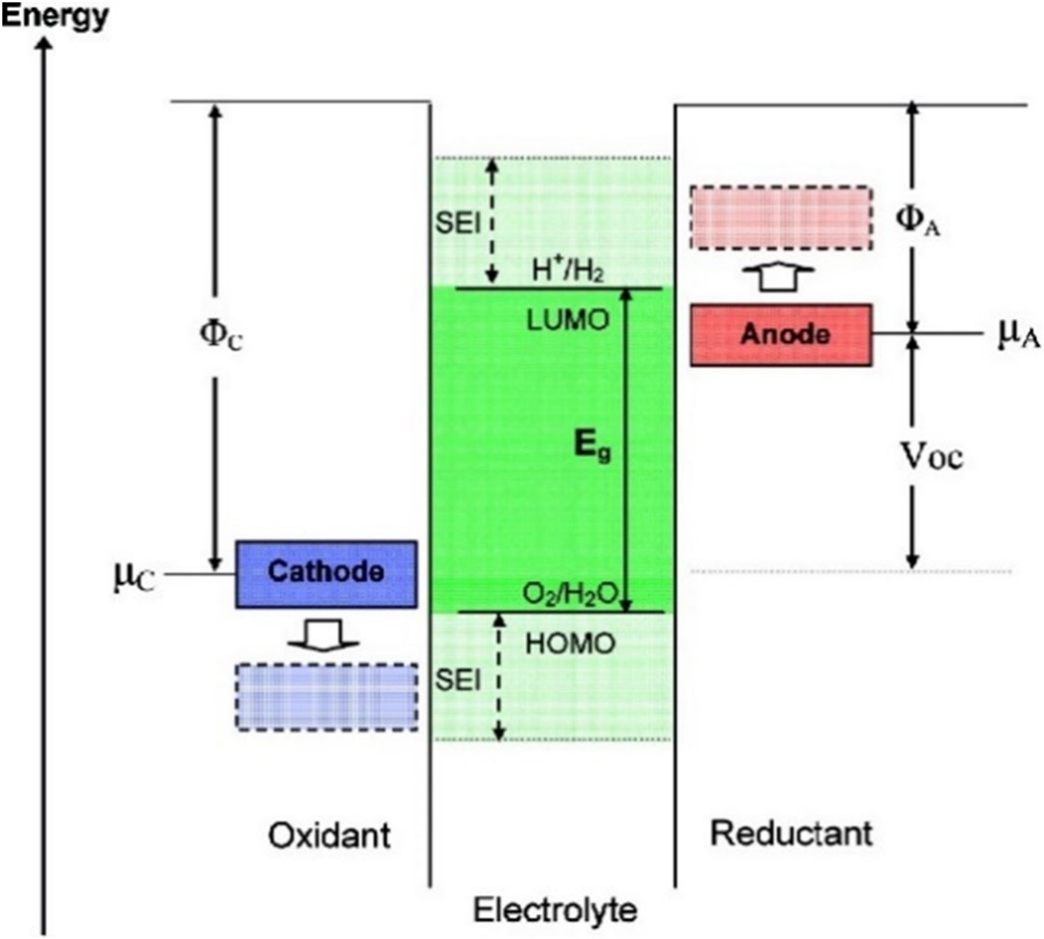
Schematic open‐circuit energy diagram of a thermodynamically stable battery having an aqueous electrolyte. Reproduced with permission.^[^ ^58^ ^]^ Copyright 2009, American Chemical Society.

In short, the formation of SEI/CEI is actually a process of redox reaction between electrolyte and electrodes. In the view of relative energy level described in **Figure** **5**, a thermodynamically stable battery system should consist of robust electrolyte and electrode materials matched with it, thereby ensuring the safe and efficient operation.^[^ ^58^ ^]^ The stability of electrolyte is determined by its highest occupied molecular orbital (HOMO) and lowest unoccupied molecular orbital (LUMO) with an electron energy gap (*E*_g_) separating them, according to frontier orbit theory. Cathode and anode have their respective Fermi levels (*ε*_F_), which can also be regarded as electrochemical potentials (*μ*), taking up distinct positions in energy map when combined with the molecular orbits of electrolyte. Typically, a cathode with high potential is located above HOMO, while anode with low potential is located below LUMO to maintain a relative equilibrium in aspect of electron energy. However, for a practical battery system, a *ε*_c_ below HOMO will cause the transmittance of electrons from electrolyte solution to cathode, oxidizing the electrolyte to generate a passivating film on cathode against further reaction. Analogically, a *ε*_a_ above LUMO will transfer electrons from anode to solution, triggering electrolyte reduction to form a SEI.^[^ ^59^ ^]^

For this reason, it is generally believed that the surface passivating films on both cathode and anode contribute to broadening the electrochemical stability window of electrolyte. Moreover, as lithium metal, silicon, and graphite, which are widely studied anode materials at present, have ultralow lithiation potentials of 0, 0.1,^[^ ^60^, ^61^ ^]^ and 0.01–0.2 V,^[^ ^62^ ^]^ respectively, currently used electrolytes tend to be reduced on negative side rather than oxidized on positive side for limited operation voltage range. Only when decomposed solvents and salts form a stable passivating SEI to broaden the electrochemical window of electrolytes can the use of anodes with low reduction potential be possible, realizing higher energy density. Otherwise, repeated breakdown of SEI in local area will constantly expose new reaction sites to electrolyte, exacerbating consumption of electrolyte and active lithium during charge/discharge cycles, and finally lead to degradation and failure of batteries.

### 2.2 The Composition and Structure of SEI on Carbonaceous Anode

**Figure 6:**
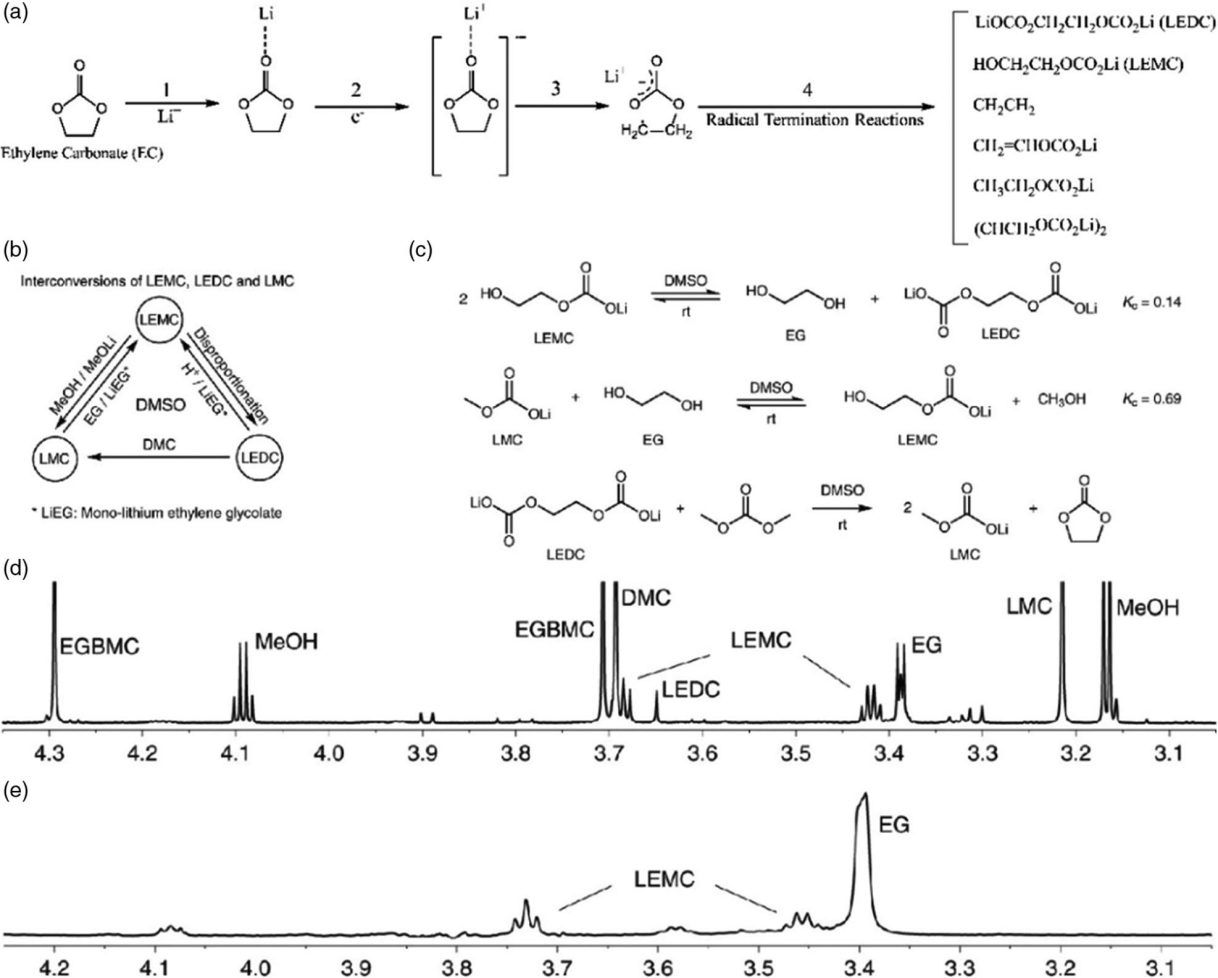
a) Reaction scheme of EC reduction a “single‐electron pathway.” The possible reaction products are listed on the right side. b,c) Interconversions between these lithium organic carbonates, in reactions with alcohols and carbonate electrolytes in DMSO. d) ^1^H NMR spectra in DMSO‐*d*_6_ collected from SEIs generated in electrolyte solutions of 1 M LiPF_6_ in EC/DMC. e) ^1^ H NMR spectra in DMSO‐*d*_6_ of SEI layers generated in the electrolyte solution of LiPF_6_/EC. Part (a): Reproduced with permission.^[^ ^64^ ^]^ Copyright 2006, American Chemical Society. Parts (b‐e): Reproduced with permission.^[^ ^65^ ^]^ Copyright 2019, Springer Nature.

With respect to SEI generated on graphite anode in LIBs, EC is one of the most common solvents used for ensuring the formation of stable SEI, and the preferential reduction of EC during initial lithiation produces major lithium ethylene dicarbonate (LEDC) and ethylene via a single‐electron route (**Figure** **6**a) in which a radical anion is formed first after one electron transference, followed by localization of negative charge on carbonyl group.^[^ ^42^, ^63^, ^64^ ^]^ Owing to the charge compensation, Li^+^ in solution will be attracted to neutralize negative charge. Finally, different radical anions assemble and combine with each other to terminate the reaction, consequently yielding a mixture of alkyl carbonates. However, Wang et al.^[^ ^65^ ^]^ recently clarified that the major organic SEI constituent on graphite is ought to be lithium ethylene monocarbonate (LEMC) instead of LEDC through elaborate spectroscopic, structural, and theoretical analysis, as researchers used to compare the SEI spectroscopic signatures with false synthetic standard sample, reported as LEDC, which was turned out to be LEMC due to reaction limitation caused by insolubility of synthetic intermediate product. In addition, as shown in Figure 6b‐c, there are complex interconversions between lithium organic carbonates and secondary reactions with alcohols in electrolyte solutions of 1 M LiPF_6_ in EC/DMC, which were further verified with solution NMR analysis (Figure 6d), showing the mixture of EC‐derived SEI products and DMC‐derived SEI products. Excluding the contributions of DMC‐related interconversions, the control experiment of SEI grown in EC‐only electrolyte indicated the presence of LEMC (Figure 6e). It could be speculated from this that LEDC has higher reactivity than LEMC toward moisture, protons, and impurities in EC, so it is instable. Even if LEDC was produced originally through the single‐electron pathway mentioned earlier, it would be converted to LEMC through reaction with protio impurities. In conclusion, despite the possible existence of LEDC in primary SEI formation stage, it is LEMC that persists in aging EC environment rather than LEDC.

For the linear dialkyl carbonates, such as DEC, EMC, and DMC, the products of reduction reactions are lithium alkyl carbonates such as LEC and LMC. Because these lithiated‐carbonate products are vulnerable to trace water in electrolyte and strong Lewis acid PF_6_^−^ in lithium salt.^[^ ^66^ ^]^ They are bound to decompose and give rise to LiF, Li_2_O, CO_2_, poly(ethylene glycolate) (PEG), lithium alkoxide, and lithium fluorophosphates (Li_*x*_PF_*y*_O_*z*_)^[^ ^67^ ^]^ adhere to the reaction formulas listed as follows.

These reactions can be simply divided into three categories: 1) classification I: conversion reactions of inorganic components. These reactions mainly involve decomposition and reduction of fluoride‐containing lithium salt, secondary reactions of by‐products from lithium salt decomposition and reduction reactions of Li^+^ with CO_2_ and H_2_O; 2) classification II: reductive decomposition of linear carbonate molecules and subsequent side reactions; and (3) classification III: decomposition reactions of LEDC, which is derived from EC, a common cyclic carbonate molecule.

**Figure 7:**
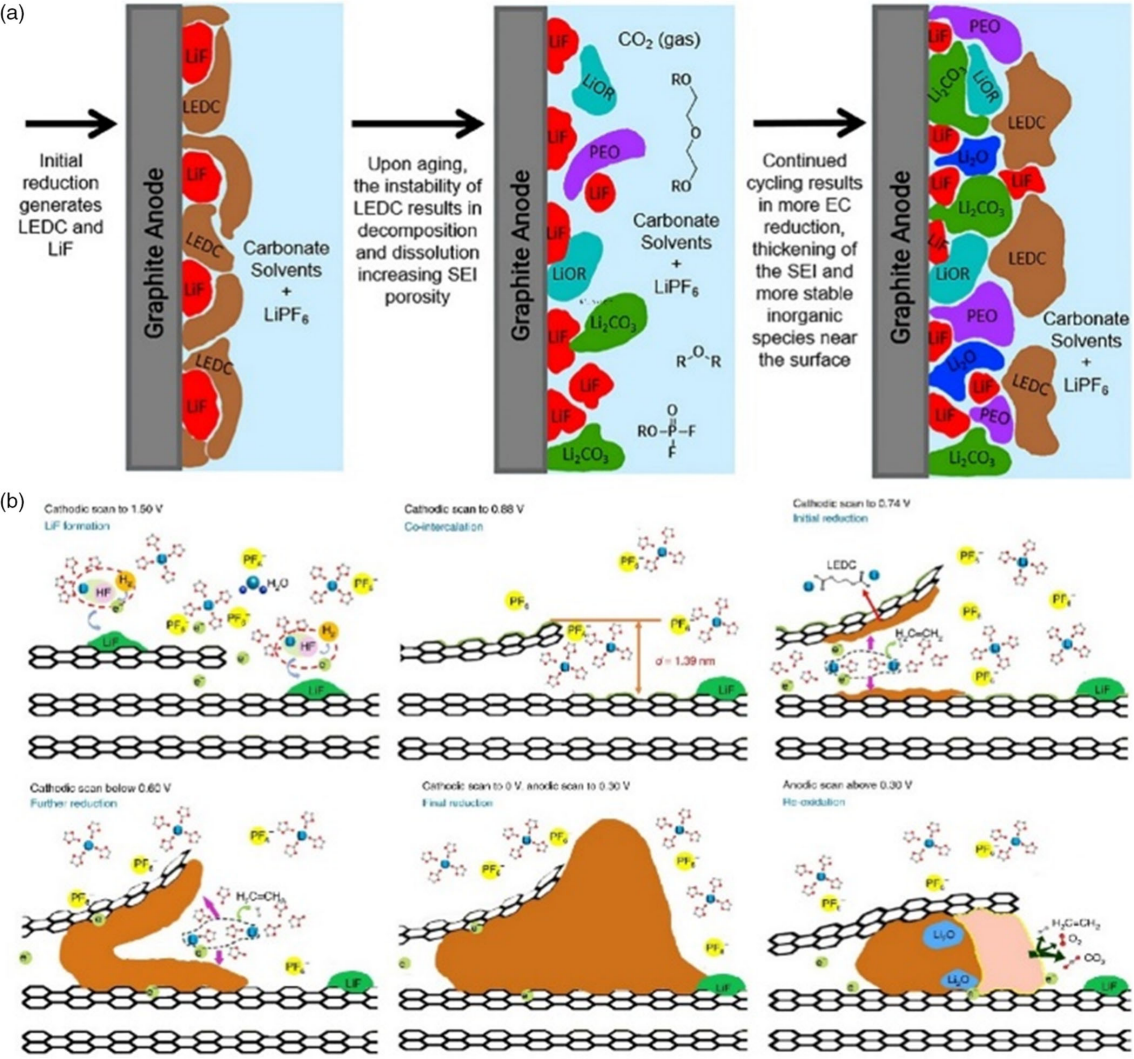
a) Schematic of the evolution of SEI formed on the graphite anode. Reproduced with permission.^[^ ^68^ ^]^ Copyright 2019, Elsevier. b) Schematic illustration of the interphasial formation chemistry during the very first lithiation on a graphic anode in a typical electrolyte composition (1.0 M LiPF_6_ dissolved in a 1:1 mixture of EC and DMC). Reproduced with permission.^[^ ^69^ ^]^ Copyright 2018, Springer Nature.

Moreover, as shown in **Table** **1**, in the view of widely used lithium salts, LiPF_6_ is subject to decomposition in the presence of moisture to produce LiF, HF, POF_3_, and other F, P‐containing species. To make a qualitative summary of the evolution of SEI on graphite anode, as shown in **Figure** **7**a, the main insoluble SEI constituents of commercial EC/dialkyl carbonate electrolytes formed on graphite anode in early stage are LEDC and LiF derived from organic solvent and inorganic lithium salts respectively. Upon aging, the hydrolyzed intermediates are prone to further decompose into Li_2_CO_3_, LiF, alkyl oxide, polyethylene oxide (PEO), and so on due to their instability, resulting in partial dissolution and porosity. In the end, more decomposition products will appear in SEI and thicken it because of serious side reactions, interconversion, and localized heat over continued cycles.^[^ ^68^ ^]^ With the aid of in situ and operando quantification characterizations conducted by the electrochemical quartz crystal microbalance (EQCM) and AFM, researchers gained mechanistic insights into the formation and electrochemistry of SEI under realistic battery conditions, and identified every link of SEI evolution more accurately.^[^ ^69^ ^]^ The schematic diagram in Figure 7b suggested that the SEI formation process began with LiF generation at 1.5 V followed by intercalating dimerization of solvated Li^+^ between graphite layers at graphite edge sites (0.88 V), and then preferential reduction of EC occurred at 0.74 V. The major reduction products of EC accumulated at lower potentials, making up the final surface structure on graphic anode with LiF and lithium alkylcarbonates as main chemical components after initial discharging. However, it is noteworthy that the lithiated reduction products of cyclic carbonate molecules were able to be reoxidized upon recharging in its nascent form, opposite to the general belief that SEI is an irreversible electrochemical inert layer, but it also seemed to be more and more difficult to be oxidized in subsequent cycles, indicating the finite reversibility and aging effects of SEI.

**Table 1:** Electrolyte reduction reactions and side reactions related to surface film formation

Categories	Reaction equations	References
Conversion reactions of inorganic components	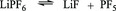	[66]
	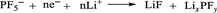	[66]
	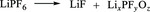	[68]
	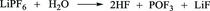	[69]
	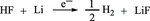	[69]
	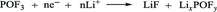	[66]
	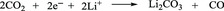	[66]
	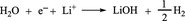	[66]
	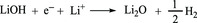	[66]
Reductive decomposition of linear carbonate molecules and subsequent side reactions	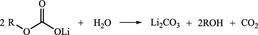	[98]
	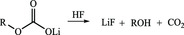	[98]
	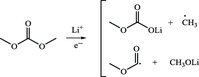	[66]
	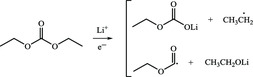	[66]
	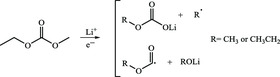	[66]
Decomposition reactions of LEDC	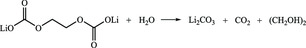	[66]
	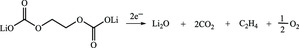	[69]
	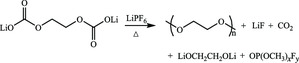	[68]

### 2.3 The Composition and Structure of SEI on Alloying‐Type Anodes

**Figure 8:**
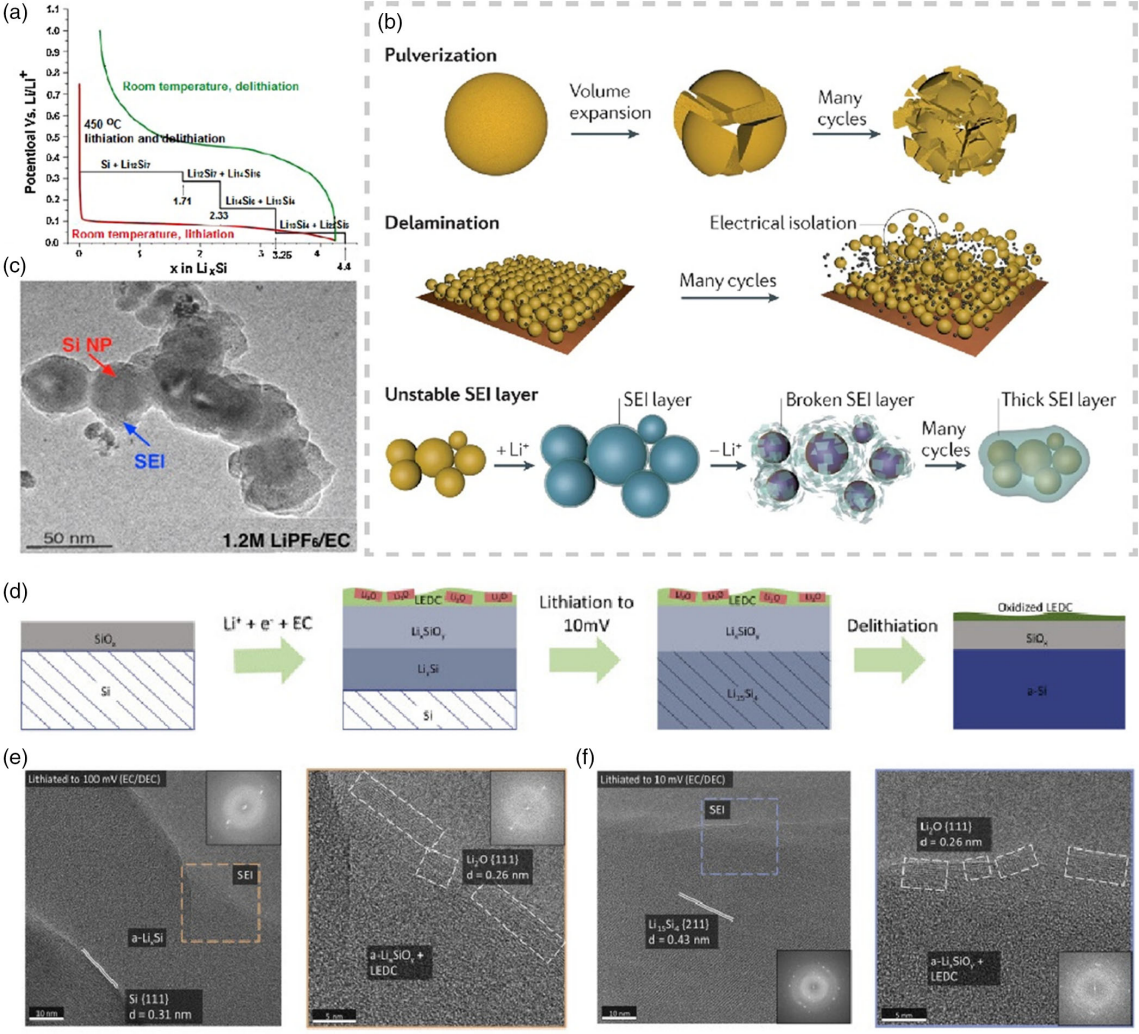
a) Si electrochemical lithiation and delithiation curve at room temperature and high temperature. Black line: theoretical voltage curve at 450 °C. Red and green line: lithiation and delithiation of crystalline Si at room temperature, respectively. b) Degradation mechanisms of Si anodes during lithiation. c) TEM images of SEI formed on BF–Si after the first 20 cycles for LiPF_6_/EC electrolyte. d) Schematic of the lithiation/delithiation of oxide‐terminated Si NW in EC/DEC electrolyte. e) High‐resolution transmission electron microscopy (HRTEM) of Si NW partially lithiated and magnified HRTEM of the SEI, to a cutoff voltage of 100 mV. f) HRTEM of Si NW fully lithiated to 10 mV, and magnified HRTEM of the SEI. Part (a): Reproduced with permission.^[^ ^70^ ^]^ Copyright 2012, Elsevier. Part (b): Reproduced by permission.^[^ ^72^ ^]^ Copyright 2016, Springer Nature. Part (c): Reproduced with permission.^[^ ^81^ ^]^ Copyright 2013, American Chemical Society. Parts (d‐f): Reproduced with permission.^[^ ^82^ ^]^ Copyright 2019, Elsevier.

Although the capacity of commercial graphite anode is close to twice that of cathode, increasing the capacity of anode still contributes to the energy density of the whole battery. Silicon is considered as the next generation anode material of high energy density LIB because of its high theoretical capacity (3578 m Ah g^−1^ for Li_15_Si_4_), low cost, environmental friendliness, and abundant reserves.^[^ ^70^, ^71^, ^72^, ^73^, ^74^ ^]^ Studies show that nanosized silicon particles transform into amorphous phase with long‐range disorder at ≈0.1 V lithiation voltage and then crystallize as Li_15_Si_4_ alloy phase around 0.05 V (**Figure** **8**a), displaying excellent lithium storage ability and low reduction potential.^[^ ^70^, ^75^, ^76^ ^]^ However, the huge volume change in silicon (320–400%) during lithiation leads to active substance shedding, SEI instability, and particle cracking or even pulverization, which are the main reasons impeding its commercialization (Figure 8b).^[^ ^72^ ^]^

For Si‐based anode materials, the reduction products of EC‐based electrolyte system in SEI are similar to that found on graphite, mainly composed of LiF, Li_2_CO_3_, Li_2_O, lithium alkyl carbonates, and Li_*x*_SiO_*y*_. Several works have been conducted to understand the structure and formation mechanism of SEI generated on silicon anode, but as far as components are concerned, conclusions vary from each other to some extent, probably stemming from different morphology of Si substrates, synthetic methods, characterization techniques, and electrolyte compositions.^[^ ^77^, ^78^, ^79^, ^80^ ^]^ For example, Lucht et al.^[^ ^81^ ^]^ reported an ex situ analysis of binder‐free Si nanoparticles (BF‐Si) via a combination of transmission electron microscopy (TEM), NMR spectroscopy, FTIR, and XPS to provide morphological and structural information about components of SEI in LiPF_6_/EC electrolyte. The results suggested that the surface of cycled BF‐Si has a layered structure with an inner SEI primarily composed of Li_*x*_SiO_*y*_ and LEDC and an outer SEI primarily composed of LEDC and LiF. As shown in Figure 8c, after 20 cycles, the SEI became thicker and amorphous, which was integrated with BF‐Si nanoparticles to form a continuous phase. Another investigation of SEI formation on silicon nanowires (Si NW) prepared by a vapor–liquid–solid (VLS) process on TEM grid was conducted via cryo‐EM,^[^ ^82^ ^]^ suggesting that in EC/DEC electrolyte, as shown in general schematic diagram in Figure 8d, lithiated (100 mV cuttoff voltage) or alloyed (deep lithiated at high state‐of‐charge, 10 mV) Si NW has an interphase with bilayer structure, consisting of an inner layer of Li_*x*_SiO_*y*_, arising from lithiation of SiO_*x*_ termination and an outer layer of LEDC and crystalline Li_2_O (Figure 8e‐f), caused by EC reduction and subsequent Li_2_CO_3_ decomposition on reductive Li_15_Si_4_. Upon delithiation, Li_2_O first converts to Li_*x*_SiO_*y*_ and is further oxidized to surficial SiO_x_, while LEDC also turns into oxidation state with sparse Li_2_CO_3_, indicating well chemical reversibility of SEI. However, the lack of SEI is unfavorable, which manifests poor cyclability. It is worth noting that LiF particles are not observed on Si NW surface via Cryo‐EM characterization nor any fluorine signals in EELS mapping, implying the absence of LiF in bulk SEI.

**Figure 9:**
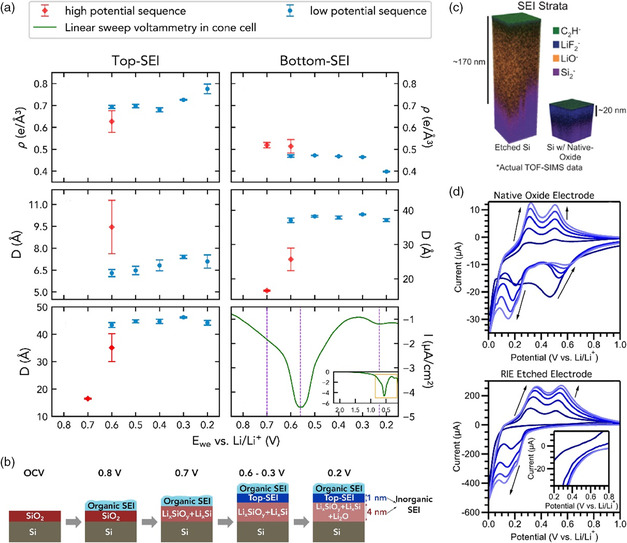
a) Best‐fit results of XRR datasets of high‐potential (red markers) and low‐potential (blue markers) sequence experiments. The electron density and thickness of the top‐SEI layer is listed on the left, while that of the bottom‐SEI layer is listed on the right. At the bottom of panel are the total thickness (left) and LSV from open circuit voltage (OCV, 2.5 V) to 0.1 V at 0.1 mV s^−1^ using a Teflon cone cell (right). b) Schematic illustration of the proposed potential‐dependent SEI growth mechanism on native oxide‐terminated Si anodes. c) Schematic of 3D SEI thickness extracted from actual TOF‐SIMS data on etched Si surface and SiO_*x*_ surface. d) Representative cyclic voltammograms using 1 m LiPF_6_ in EC/DEC (1:1 by wt%) and (100) silicon wafer working electrodes with a native oxide and with a reactive ion etching (RIE) etched surface. Parts (a,b): Reproduced with permission.^[^ ^83^ ^]^ Copyright 2019, Elsevier. Parts (c,d): Reproduced with permission.^[^ ^84^ ^]^ Copyright 2014, American Chemical Society.

To shed light on the specific potential range of SEI formation and evolution process on native oxide‐terminated Si, the combination of in situ surface‐sensitive electrochemical characterization techniques with nanoscale resolution and suitable models for predicting equilibrium phase are quite challenging but urgently needed. Toney et al.^[^ ^83^ ^]^ conducted an ingenious measurement combining in situ synchrotron X‐ray reflectivity (XRR: exclusively sensitive to inorganic fraction due to its strong reflection contrast), linear sweep voltammetry (LSV), ex situ XPS, and first‐principles calculations for crystalline Si (001) wafer electrode with little SiO_2_ on surface to characterize evolution process of SEI structure along with potential change in LP30 (1 m LiPF_6_ in EC/DMC = 1:1 by weight) carbonate‐based electrolyte. The normalized XRR pattern extracted from Si wafer held at different potential revealed inorganic SEI composed of two parts, among which the SEI at the bottom nucleated at 0.7 V and its density decreases at 0.2 V, indicating low‐density Li_2_SiO_*y*_/Li_*x*_Si and Li_2_O, while the top SEI starts to form at 0.6 V with increasing density, indicating the existence of LiF (**Figure** **9**a). Coupling with other spectroscopic data and density functional theory (DFT)‐based simulation results, a model of SEI changing with voltage has been set up (Figure 9b). Specifically, the organic SEI layer is expected to form at potential above 1.5 V. Initial Li_*x*_Si appears before 0.7 V due to Li^+^ diffusion into bulk Si. No inorganic SEI is observed until the potential decreases to 0.7 V, forming bottom low‐density Li_*x*_SiO_*y*_ layer, which results from the lithiation of SiO_2_. Starting form 0.6 V, LiPF_6_ electrochemical reduction yields a top‐layer SEI mainly composed of LiF. In the last stage, Li_*x*_SiO_*y*_ in bottom SEI is further lithiated to form Li_2_O at 0.2 V, leading to lower electron density.

In fact, it is inevitable that the surfaces of silicon particles are covered with a layer of oxide when exposed to oxygen, and so the works mentioned earlier are not targeted to SEI formed on intrinsic pure Si but SiO_*x*_. Thus, the investigations about SEI on Si surfaces without oxide are imperative to make a comparison with native oxide‐terminated one. Recently, Schroder et al.^[^ ^84^ ^]^ reported reactive ion etched silicon wafer in standard 1 m LiPF_6_ EC/DEC (1:1 by weight) electrolyte as a model system for insights of the effects of surface oxide on SEI formation, composition, and structure. As shown in cyclic voltammetry curves in Figure 9d, the etched sample had no extra reduction peak in the range of 0.47–0.59 V, which was ordinarily indexed to partial lithiation of the native‐oxide layer. Furthermore, the time‐of‐flight secondary ion mass spectrometry (TOF‐SIMS) depth profiles showed a much thicker SEI (≈170 nm) of etched lithiated silicon electrode enriched with inorganic species, primarily Li_2_O, which was speculated to source from carbonate solvents or organic SEI products (Figure 9c). Meanwhile, the lithiated oxide‐native Si had a relatively compact SEI that enables the formation of stable LiF, P_*x*_O_*y*_F_*x*_, and organic components without Li_2_O. Based on the understanding of oxide passivation mechanism and silicon surface charge transfer behavior, the difference of SEI thickness and ingredient was attributed to the synergic effect that is owing to rapid electron transport caused by high conductivity of etched Si surface and sluggish kinetics of lithiation as well as unrestrained mechanical changes, allowing massive side reactions to happen. The work holds that the reversible formation of Li_2_O on etched Si surface is detrimental to cell operation because it implies ceaseless consumption of electrolyte and emission of gases, resulting in porous nanostructure, which will be aggravated under the high‐rate condition. In addition, it is also expected that planar amorphous Si deposited by magnetron sputtering in argon atmosphere could be a reliable platform to study SEI on oxygen‐free surface and quantify the capacity loss due to SEI formation owing to the regular geometry.^[^ ^77^ ^]^ Unlike etched crystalline Si, SEI formed on planar amorphous Si wafer (1 m LiPF_6_ in EC:DEC:DMC = 1:1:1 as electrolyte) consists of ROCO_2_Li, LiF, and Li_*x*_PO_*y*_F_*z*_ at constant 0.2 V discharge cutoff voltage, and the product concentration increases with hold time, indicating the electrochemical instability of SEI formed on oxygen‐free silicon surface once again.

Above all, the tremendous volume deformation and unstable SEI film of silicon anode material are the intrinsic problems, calling for more advances in particle size optimization, surface modification, morphology and structure design,^[^ ^85^ ^]^ electrolyte additives,^[^ ^86^, ^87^ ^]^ binders, and so on to regulate the properties of SEI and alleviate mechanical strains.^[^ ^25^ ^]^ In addition, the prelithium compensation strategy along with amorphous silicon alloy and nanosilicon–carbon composite material design should be taken into consideration as well to address low initial CE and bring about excellent cycling performance.^[^ ^88^, ^89^, ^90^ ^]^

Similar to silicon‐based anode, another widely studied alloying‐type anode is Sn‐based alloy due to its low average potential (≈0.6 V) and high specific capacity of ≈994 mAh g^−1^ more than twice as much as graphite, corresponding to Li_4.4_Sn.^[^ ^91^ ^]^ Unfortunately, Sn anode also suffers from large volume deformation (≈260%) in conventional EC‐based electrolyte so that the SEI layer is subject to rupture and dissolve without abundant protection for substrate, leading to irreversible electrolyte decomposition and rapid capacity fade. In situ AFM characterization on Sn anode during cyclic voltammetry (CV) cycles demonstrated that the surface film initializes at ≈2.8 V (vs Li^+^/Li), following with gradually changes at 0.7–2.5 V potential range, where continuous deformation happens and then compromises the reversible capacity.^[^ ^92^ ^]^ It is generally acknowledged that the SEIs formed on Sn nanoparticle electrodes in standard electrolyte of 1.2 m LiPF_6_ in EC/DEC consist of lithium alkyl carbonates (ROCOOLi), Li_2_CO_3_, LiF, and Li_*x*_PO_*y*_F_*z*_. However, the distribution of organic components across the SEI on Sn is homogenous as thickness, which is quite different from the bilayer structure featured with inner/outer differences observed on Si.^[^ ^93^ ^]^ At present, the modification of Sn alloy anode is mainly based on the principle of structural design to quantitative dope Sn oxide for improved cycle performance. As a result, amorphous Sn‐based composite oxides^[^ ^94^ ^]^ and SnMC alloy (M = Ti, V, Co)^[^ ^95^, ^96^, ^97^ ^]^ emerges as promising candidates for replacement of Si‐based anodes.

### 2.4 The Composition and Structure of SEI on Lithium Anode

**Figure 10:**
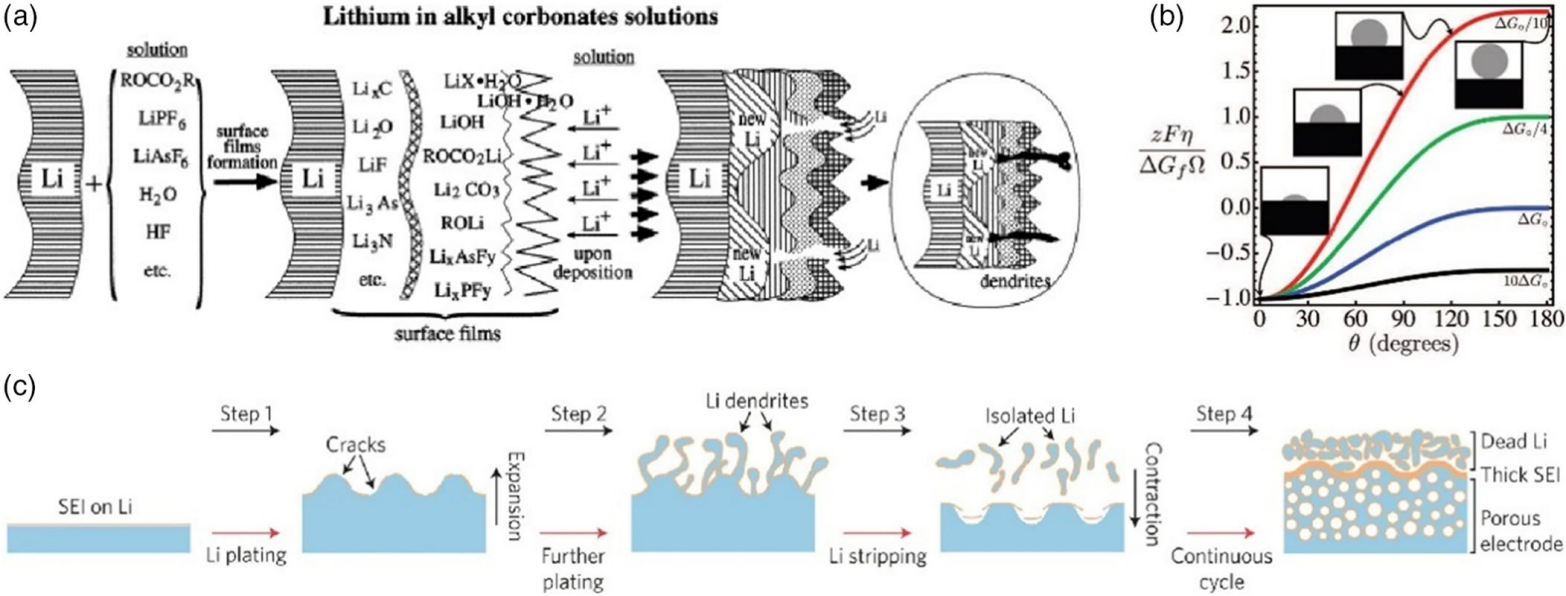
a) A schematic illustration of surface film formation on lithium electrodes in alkyl carbonates. Reproduced with permission.^[^ ^66^ ^]^ Copyright 2000, Elsevier. b) Effect of precipitate contact angle on the normalized critical overpotential for selected critical free energies of transformation, $\Delta G_{c}$. $\Delta G_{o}=\frac{16\pi \gamma_{\text{NE}}^{3}}{3\Delta G_{f}^{2}}$ is the critical Gibbs free energy of transformation for a homogeneously nucleated precipitate, i.e., for *θ* = 180° and *η* = 0. Reproduced with permission.^[^ ^101^ ^]^ Copyright 2013, IOP Publishing. c) Schematic showing the Li stripping/plating process. Reproduced with permission.^[^ ^100^ ^]^ Copyright 2017, Springer Nature.

As mentioned earlier, owing to the limited potential window of available electrolytes and highly negative electrochemical potential of Li^+^/Li, the formation of SEI film on lithium anode is virtually inevitable. It is well recognized that the understanding of SEI film on lithium anode is on the basis of pioneering works by Peled^[^ ^27^, ^28^, ^31^, ^47^ ^]^ and Aurbach,^[^ ^42^, ^43^, ^44^, ^63^ ^]^ who have not only depicted the physiochemical properties of SEI but also modeled its structure according to identified compositions. Among various nonaqueous solvents and lithium salts with perfluorinated anions (XF_6_^−^, X = P, B, As, Tf) system, LiPF_6_‐based electrolyte is considered as an optimum choice because it has rapid reactive kinetics with lithium to yield a more efficient and protective film, which could be partially ascribed to LiF in SEI, with minimum consumption of electrolyte and active lithium.^[^ ^98^ ^]^ Therefore, a commercial LiPF_6_^−^based carbonate electrolyte is the most common and basic model system for studying the formation of SEI on lithium. In general, apart from insoluble inorganic like Li_2_CO_3_, the main reduction products of solvent precipitated on lithium anode are lithium alkoxide, lithium alkyl carboxylate, and lithium alkyl carbonate originated from ethers, esters, and alkyl carbonate, respectively.^[^ ^66^ ^]^ For example, cyclic carbonate solvent molecules such as EC and PC are prone to be reduced to LEDC and lithium propylene dicarbonate (LiOCO_2_CH(CH_3_)CH_2_OCO_2_Li, LPDC) separately at ≈0.7 V,^[^ ^99^ ^]^ while for linear molecules such as DMC, DEC, and ethyl methyl carbonate (EMC), their respective derived alkoxides (CH_3_OLi, CH_3_CH_2_OLi) and lithiated alkyl monocarbonates (CH_3_OCO_2_Li, CH_3_CH_2_OCO_2_Li) are the more common products due to quite different free radical mechanism of nucleophilic reaction.^[^ ^42^, ^64^ ^]^ As to PF_6_^−^ preexisting in Li^+^ solvation shed, the ingredients from the anion reductive composition on lithium metal surface are LiF‐ and Li_*x*_PF_*y*_‐type species. In addition, even if the electrolyte is rigorously dried, the existed trace water is still unavoidable to hydrolyze fractional alkyl carbonate or even penetrate toward active material, yielding Li_2_CO_3_, LiOH, and Li_2_O. Meanwhile, the tiny amount of carbon dioxide that arises from solvent oxidation on cathode side and carbonate hydrolysis will also conversely stimulate the production of Li_2_CO_3_.^[^ ^66^ ^]^ Therefore, in the light of these compositional understandings, a diagram of surface film formation on lithium electrode has been built up (**Figure** **10**a), where a SEI with multilayered structure has an inner compact part and an outer porous part due to the solubility equilibrium of surface species and the increase in reactive selectivity as SEI becomes thicker.

When it comes to the failure process of lithium metal surface film, the growth of lithium dendrite and large volume expansion are two major factors that cannot be ignored. As shown in Figure 10c, the heterogenous Li^+^ conductivity among SEI domains and high current density will induce the fluctuation of surface film morphology during Li plating process, finally cracking fragile SEI and exposing fresh Li to electrolyte.^[^ ^100^ ^]^ As Li‐ion flux increases at the fracture, the nucleation of lithium metal tends to be strengthened at these rough areas with decreasing thermodynamic curvature radius, leading to reduced critical overpotential (Figure 10b).^[^ ^101^ ^]^ Consequently, lithium dendrites prefer to grow on the tips while extra electrolyte consumption also emerges to create passivation layers on fresh surfaces. Further breakdown of SEI will take place during subsequent Li stripping process, which disconnects partial dendrites from substrates or current collectors, leading to “dead” lithium.^[^ ^48^, ^102^ ^]^ After repeated plating/stripping, excessive dead Li, a thick SEI and porous surface accumulate on lithium metal electrode, eventually resulting in capacity fading along with a series of safety hazards such as increased internal resistance, short circuit, local overheating, and gas production.

Therefore, to fulfill comprehensive excellent electrochemical performance and eliminate safety risks in nonaqueous LMBs, a robust SEI film is expected to construct in accordance with the following few principles:^[^ ^98^ ^]^ 1) thorough electronic insulation; the thickness of SEI is ought to exceed the electron tunneling range to suppress the movement of electrons from bulk phase to solution. 2) High ionic conductivity; the transfer number of lithium ions is as close to a unit as possible ($t_{{\text{Li}}^{+}}\approx 1$) while anionic transfer number should approach to zero, ensuring a small enough resistance when Li^+^ passes through SEI film. 3) Morphological and chemical structural stability; that means the morphology and chemical structure does not change with cycles. 4) Well binding with active substances; it may not exfoliate during cycling. 5) Favorable mechanical properties; it has the ability to accommodate the volume change in the process of charging and discharging without cracking or even pulverization. 6) Splendid electrochemical stability and thermal stability; the main components of SEI are insoluble in electrolyte in a wide voltage range, and once formed and stabilized, they will not participate in redox reaction any more within the working potential range. In addition, SEI films need to be stable in a broad temperature range without decomposition and highly crystallization.

## 3 The Fundamental Physicochemical Properties of Fluorides

Previous studies offer abundant information with spectral resolution and atomistic spatial precision about the chemical components of the SEI, which has mostly confirmed the presence of LiF arising from decomposition of perfluorinated lithium salts or fluorinated electrolyte components. Moreover, many studies in recent years also rationalized the beneficial function of LiF by its unique physicochemical properties capable of passivating anode to achieve improved cyclability.^[^ ^103^ ^]^ Specifically, the passivation ability of LiF stems from its large bandgap, low electronic conductivity, low carrier concentration, high interfacial energy, and excellent electrochemical stability compared with other inorganic ingredients in SEI such as Li_2_CO_3_ and Li_2_O. Driven by the positive effect on dendrite inhibition, adjustment of interfacial Li^+^ diffusion, acid erosion resistance, and so on, a LiF‐rich artificial interphase has been intensely pursued to bring about performance beyond conventional organic electrolyte‐derived SEI, especially in the field of LMBs, as researchers take it for granted that LiF is responsible for better lithium deposition behavior and cycling performance. Therefore, it is necessary to make a special introduction to the characteristics and functions of lithium fluoride in this part of section.

**Figure 11:**
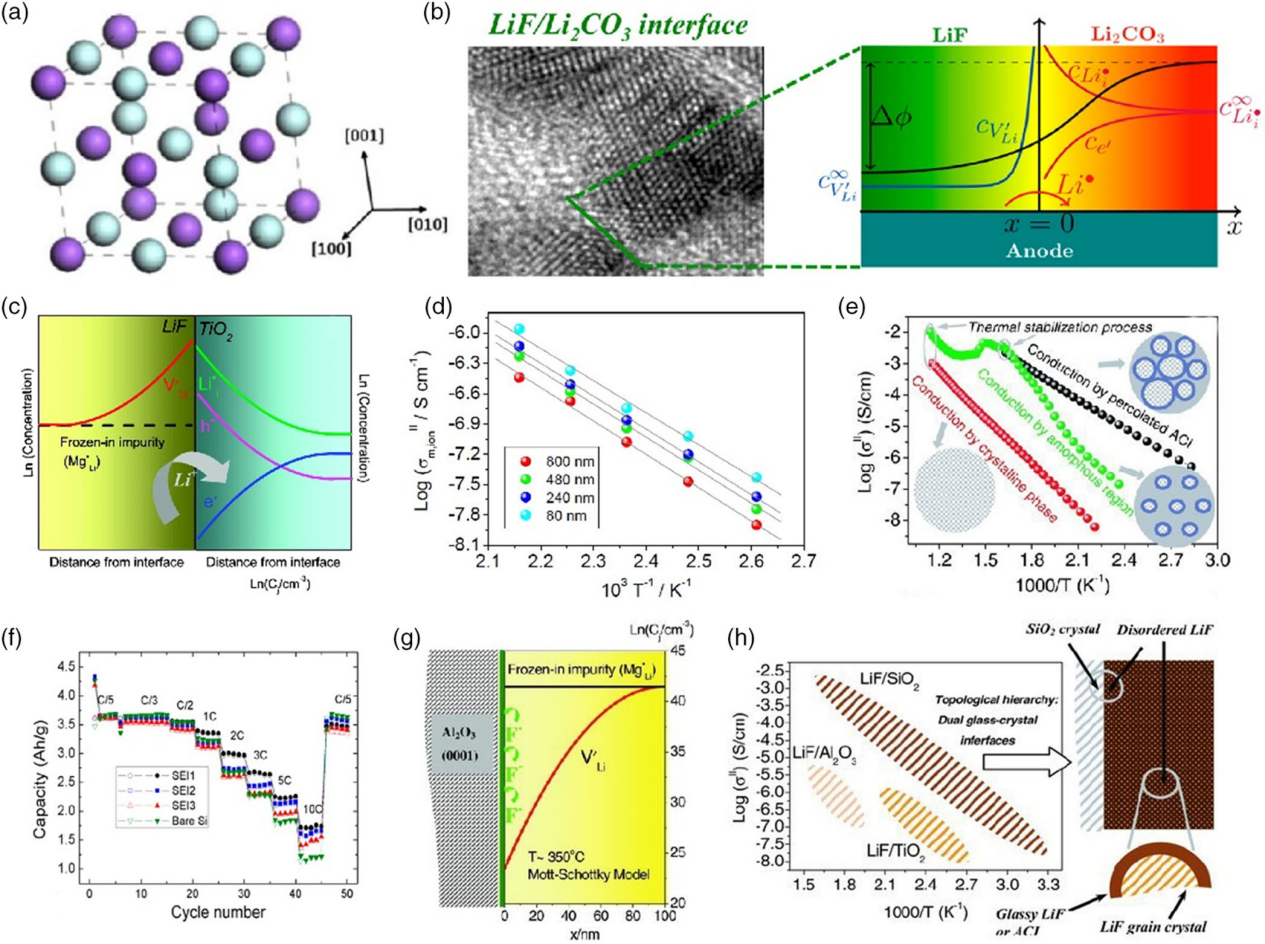
a) The atomic structure of LiF (cubic, space group: Fm‐3 m). b) Schematic figure showing the defect reaction and defect distribution near the LiF/Li_2_CO_3_ interface. c) Overview defect chemistry schemes and carrier concentration profiles of LiF/TiO_2_ interface. d) Arrhenius plots of LiF thin films with various thicknesses (80–800 nm). e) Arrhenius plots of LiF thin films (417 nm) during different heating–stabilization–cooling cycles with corresponding Li^+^‐migration paths. f) Rate capability of Si electrodes with different artificial SEI coatings; SEI1 has the highest LiF content. g) Concentration profile of the depleted lithium vacancy (V′_Li_) as a function of distance from LiF–sapphire interface at 350 °C based on Mott–Schottky model. h) Arrhenius plot regions of LiF–SiO_2_, LiF–TiO_2_, and LiF–Al_2_O_3_ systems. Part (a): Reproduced with permission.^[^ ^107^ ^]^ Copyright 2016, Elsevier. Parts (b,f): Reproduced with permission.^[^ ^111^ ^]^ Copyright 2016, American Chemical Society. Parts (c,d): Reproduced with permission.^[^ ^112^ ^]^ Copyright 2012, American Chemical Society. Parts (e,h): Reproduced with permission.^[^ ^113^ ^]^ Copyright 2012, Wiley‐VCH. Part (g): Reproduced with permission.^[^ ^115^ ^]^ Copyright 2011, Wiley‐VCH.

As shown in **Figure** **11**a, LiF has a rock‐salt structure that can be indexed to a cubic symmetry with Fm‐3 m space group, while their lattice parameter is calculated theoretically to be almost 4.00 Å with a large bandgap of 8.70 eV, which proves itself as an electronic insulator.^[^ ^104^, ^105^, ^106^ ^]^ For further verification, the electron tunneling barriers of three inorganic insulating SEI components (LiF, Li_2_CO_3_, Li_3_PO_4_) on lithium metal surface are qualitatively calculated by optimized DFT approaches (DFT/HSE06).^[^ ^107^ ^]^ Based on bulk perfect crystal model without regard to fluctuations caused by quantum size effect, the bandgap of LiF is 10.8 eV while the universal generalized gradient approximation (GGA) calculations provide an underestimated value of 8.52 eV, showing a larger discrepancy with experimental value (14.1 eV). Nevertheless, a reasonable comparison among these SEI components is accessible and the electronic tunneling barrier follows the same order as bandgap of LiF > Li_3_PO_4_ > Li_2_CO_3_ regardless of the adopted calculation methods. The critical thickness to block leaking electrons is another pivotal measurement for screening appropriate coating layers to modify anodes. A LiF layer with a thickness of 1.6–2 nm, thinner than other counterparts, is suggested to be sufficient. Considering the strain effect caused by volume change under different state of charges, it is clear that larger average distances between anions are usually accompanied by smaller bandgaps, leading to the reduction in tunneling barrier and a thicker SEI. Therefore, from this point of view, LiF is suitable for mitigating irreversible capacity loss as an excellent self‐limiting electronic insulator.

The pure crystallized LiF phase is generally considered to be unfavorable for Li^+^ migration as it has poor ionic conductivity (≈10^−13^ S m^−1^),^[^ ^108^ ^]^ low defect concentration, and high transport barrier. Unlike Li_2_CO_3_ and Li_2_O with fast ion diffusion in “compact” SEI, strongly localized holes created by lithium vacancies in LiF lattice make few contributions to lithium migration. The ranking of energy barrier of lithium migration for three important inorganic compounds in SEI is LiF > Li_2_CO_3_ > Li_2_O, which is stimulated using nudged elastic band method.^[^ ^104^ ^]^ However, naturally formed SEI is full of grain boundaries and heterogeneous structures due to its multicomponents coexisting features and complex mosaic morphology. In a view of defect chemistry, the ion transport performance of LiF is likely to be enhanced by interfacing with other SEI nanodomains such as relatively more ionic conductive Li_2_CO_3_ (≈10^−8^ S m^−1^)^[^ ^109^, ^110^ ^]^ because of the formation of space‐charge layer.^[^ ^108^, ^111^, ^112^, ^113^ ^]^ Xiao et al.^[^ ^111^ ^]^ designed a SEI model system with specific proportion of LiF and Li_2_CO_3_ on silicon thin film electrodes to verify the facilitation to Li^+^ transport. According to the computed results of the formation energy of lithium diffusion carriers by DFT, Li^+^ interstitials (${\text{Li}}_{i}^{\cdot }$) are the dominant ionic carriers in Li_2_CO_3_ while Schottky pairs (Li^+^ and F^−^ vacancy pair, SP, Kröger–Vink notation: $V_{\text{Li}}^{\text{'}}{+V}_{F}^{\text{•}}$)^[^ ^114^ ^]^ play a leading role in LiF. Considering all possible defect reactions across LiF/Li_2_CO_3_ interface, it is found that only Li^+^ migration from LiF to Li_2_CO_3_ is energetically favorable as the sum chemical potential of ${\text{Li}}_{i}^{\cdot }$ in Li_2_CO_3_ and $V_{\text{Li}}^{\text{'}}$ in LiF is negative. This Li^+^ migration direction forms a $V_{\text{Li}}^{\text{'}}$ in LiF and a ${\text{Li}}_{i}^{\cdot }$ in Li_2_CO_3_, expressed by the following reaction: ${\text{Li}}_{\text{Li}}(\text{LiF})\Leftrightarrow {\text{Li}}_{i}^{\cdot }({\text{Li}}_{2}{\text{CO}}_{3}){+V}_{\text{Li}}^{\text{'}}(\text{LiF)}$, leading to the accumulation of point defects in both side, which creates a space charge layer with electrostatic potential across the interface (Figure 11b). Such potential field results in the gradient enrichment of Li^+^ interstitials and depletion of electrons. Therefore, the ionic conductivity can be greatly improved by introducing heterogeneity to increase the concentration of ionic carriers at interfaces. As a result, an improved rate capability of Si electrode has been achieved with the mixture Li_2_CO_3_/LiF coating (Figure 11f), implying beneficial function of reasonable fluorinated interface engineering.

In fact, other studies on addition of insulating oxide into LiF have also suggested that the ionic and electronic redistribution effect could be ionically controllable for sake of desired ionic/electronic quality through selecting suitable additives and building up solid/solid interfaces. Li et al.^[^ ^112^ ^]^ verified redistribution of Li^+^ at contact LiF/TiO_2_ interface using both ion‐ and electron‐blocking measurement architectures containing penetrated Pt microelectrodes and the results demonstrated that lithium vacancy conductivity increased in LiF side corresponding to compensating excess charge on TiO_2_ side (Figure 11c). The charge accumulation in TiO_2_ consumes excess electrons and gives rise to transition from n‐ to p‐type conductivity that is consistent with space charge model. It is worth noting that at the same temperature, the conductivity gradually varies with thickness of LiF inversely, but activation energies calculated from linear fitting waves around 0.65 eV (Figure 11 d), which shows that it is ion carrier accumulation, not alternative Li^+^ mobility that should be responsible for higher conductivity. Similar phenomenon can also be found in the case of LiF/SiO_2_, but the interconnection effect of heterogeneous interfaces is specially highlighted.^[^ ^113^ ^]^ The silica substrates served as dopant and glass network former during initial bombardment film formation stage promotes the formation of amorphous phase surrounding crystallized LiF zones, so as to build percolating interfaces (ACI) between the two phases integrally. The thickness and sintering temperature of lithium fluoride not only regulate the degree of amorphization but also directly influence grain size, enabling a great majority of percolated ACI and optimal ionic conductivity of ≈6 × 10^−6^ S cm^−1^ at 50 °C with low activation energy of 0.55 eV (Figure 11e), three orders of magnitude higher than bulk single‐crystal LiF film. Therefore, it can be concluded that the Li^+^ conductivity of LiF would be remarkably influenced by local microstructure.

Nonetheless, not all microstructure effects are positive for Li^+^ conductivity of LiF because a negative space‐charge potential will deplete Li^+^ vacancies, leading to charge carrier concentration unfavorable for migration as depicted in LiF contacted with sapphire (mainly Al_2_O_3_).^[^ ^115^ ^]^ In contrast to analogical LiI/Al_2_O_3_^[^ ^116^ ^]^ or LiF/TiO_2_ and LiF/SiO_2_ interfaces (Figure 11h), preferential adsorption of F^−^ rather than Li^+^ at Al_2_O_3_ surface takes place to compensate the excess positive charge brought by impurities (magnesium defect), and as a result, $V_{\text{Li}}^{\text{'}}$ drops sharply close to the interfaces (Figure 11g). Therefore, selecting the ion conductor with appropriate chemical potential and removing adverse impurities are of great significance to improve the defect chemistry of lithium fluoride and Li^+^ transport performance.

In conclusion, current researches on Li^+^ transport mechanism in bulk phase or interface of LiF mostly stay on the level of theoretical calculation and model deduction of charge carriers, lacking convincing visual experimental evidence in reality, and whether these results conform to the complicated practical systems is still in doubt. Even so, some guidance can be provided with these computed and electrochemical data to understand the exclusive function of LiF in electronic and ionic properties of SEI. As generally believed that LiF is an insulator of ions and electrons, the improved electrochemical performance brought by F‐containing SEI should be attributed to the enhancement of Li^+^ diffusion at interfaces and grain boundaries because LiF often exists in the form of particles. Furthermore, recent advances in LMBs demonstrated that compared with in situ natural SEI defined by thermodynamics in commercialized electrolytes, a LiF‐rich artificial SEI possessed higher surface energy, faster diffusion rate, and lower diffusion barrier of Li^+^ and thus rendered a dendrite‐free lithium anode, leading to better stability. Similar effect will be taken even if LiF is only used as electrolyte additives.^[^ ^117^ ^]^ Meanwhile, when it comes to surface modification of cathodes for LIBs, inspired by the physiochemical properties of LiF, metal fluorides have been explored to improve the interfacial compatibility between cathode materials and electrolyte. It is highlighted that differing from the commonly used oxide coating materials, the high corrosion resistance of fluoride makes it possible to block the notorious side reaction loop of continuous HF production triggered by trace water.^[^ ^118^ ^]^

## 4 Metal Fluoride Passivation Coatings for Cathodes

Metal fluorides have been widely studied as coating materials for state‐of‐the‐art cathodes of LIBs, such as layered oxides (including LiCoO_2_, Ni‐rich, and Li‐rich cathodes) and spinel‐type oxides in light of their excellent stability at high voltage as well as chemical stability against acid species. Fluoride coatings often act as physical barriers to avoid direct contact between electrodes and nonaqueous electrolytes in terms of role and function because of their electrochemical inertness, which makes for mitigating detrimental unwanted surface reactions and delaying irreversible phase transitions especially in high charged state.^[^ ^119^, ^120^ ^]^ In addition, another view proposed by several researchers to explain the role of fluoride coatings is that the coating materials may react with the surface of substrate or even diffuse into the surface lattice vacancies, depending on categories of cathodes, to facilitate the ionic charge at interphase.^[^ ^121^, ^122^, ^123^, ^124^, ^125^ ^]^ By this way, it is possible to form a new compatible interphase that results from cation/anion doping driven by sintering process, with better structural stability. However, with the aim of optimizing the electrochemical performance, the thickness, morphology, elemental composition, and processing procedure of fluorides need to be taken into careful consideration for desirable surface stabilization effect and satisfactory charge transfer. In this regard, it is reasonable to expect that a thin and uniform fluoride coating with moderate ionic/electronic conductivity and feasible processing be urgently required. In recent years, some works about different kinds of fluoride coating materials have been reported, as shown in **Table** **2**. We summarized the representative works in fluoride coatings on various cathode surfaces, which mainly focused on their voltage windows for measurement, reversible capacity, cycling stability, and preparation methods.

**Table 2:** A summary of LIB cathode materials coated with metal fluorides concentrating on voltage window, reversible capacity, cycling stability, and preparation methods

Chemical formula	Voltage [V]	Practical capacity [mAh g^−1^]	Capacity retention	Preparation
LiCoO_2_ (LCO)	–	–	–	–
LCO@AlF_3_^[^ ^134^ ^]^	3.0–4.5	182.13 (20 mA g^−1^, 30 °C)	98% (50 cycles)	Wet chemistry, 400 °C 5 h, N_2_
LCO@LAF (Li, Al, F‐based hybrid treatment)^[^ ^149^ ^]^	3.0–4.6	208.7 (27.4 mA g^−1^, RT)	81.8% (200 cycles)	Hydrothermal, 500 °C 6 h, Ar
LCO@AlW_*x*_F_*y*_^[^ ^155^ ^]^	2.5–4.4	∼165 (20 mA g^−1^, RT)	99% (50 cycles)	ALD, 200 °C, N_2_
LCO@MgF_2_^[^ ^214^ ^]^	3.0–4.5	≈180 (54.8 mA g^−1^, RT)	80% (50 cycles)	Wet chemistry, 400 °C 5 h, Air
LiNi_0.5_Mn_1.5_O_4_ (LNMO)	–	–	–	–
LNMO@LiF (fluoride source: Hfac)^[^ ^154^ ^]^	3.5–4.85	≈136 (0.1 mA cm^−2^, RT)	≈67% (100 cycles)	ALD, 220 °C, N_2_
LNMO@LiF (fluoride source: TiF_4_)^[^ ^154^ ^]^	3.5–4.85	≈115 (0.1 mA cm^−2^, RT)	≈72% (100 cycles)	ALD, 220 °C, N_2_
LNMO@MgF_2_^[^ ^136^ ^]^	3.5–4.95	≈80 (0.1 mA cm^−2^, RT)	≈86% (140 cycles)	ALD, 275 °C, N_2_
LiNi_1–*x*–*y*_Co_*x*_Mn_*y*_O_2_ (NCM) or LiNi_1–*x*–*y*_Co_*x*_Al_*y*_O_2_ (NCA)	–	–	–	–
LiNi_1/3_Co_1/3_Mn_1/3_O_2_@CaF_2_^[^ ^130^ ^]^	2.5–4.6	161.0 (40 mA g^−1^, RT)	93.5% (65 cycles)	Wet chemistry, 650 °C 6 h, air
LiNi_0.8_Co_0.1_Mn_0.1_O_2_@CaF_2_^[^ ^131^ ^]^	2.7–4.3	148.2 (275 mA g^−1^, 25 °C)	85.4% (200 cycles)	Wet chemistry, 500 °C 5 h, air
LiNi_0.8_Co_0.1_Mn_0.1_O_2_@LiF^[^ ^215^ ^]^	2.8‐4.3	≈172 (2 C, RT)	82.2% (200 cycles)	Wet chemistry, 300 °C 3 h, air
LiNi_1/3_Co_1/3_Mn_1/3_O_2_@AlF_3_^[^ ^135^ ^]^	3.0–4.5	173 (80 mA g^−1^, RT)	93% (50 cycles)	Wet chemistry, 400 °C 5 h, N_2_
LiNi_0.8_Co_0.1_Mn_0.1_O_2_@AlF_3_^[^ ^132^ ^]^	3.0–4.3	196 (100 mA g^−1^, 30 °C)	92.7% (60 cycles)	Wet chemistry, 400 °C 5 h, O_2_
LiNi_0.5_Co_0.2_Mn_0.3_O_2_@LaF_3_^[^ ^133^ ^]^	3.0–4.8	≈216 (200 mA g^−1^, RT)	74.1% (50 cycles)	Wet chemistry, 400 °C 3 h, air
LiNi_0.5_Co_0.2_Mn_0.3_O_2_@MgF_2_^[^ ^133^ ^]^	3.0–4.8	≈203 (200 mA g^−1^, RT)	83.2% (50 cycles)	Wet chemistry, 400 °C 3 h, air
LiNi_0.5_Co_0.2_Mn_0.3_O_2_@AlF_3_^[^ ^140^ ^]^	2.5–4.3	139.8 (100 mA g^−1^, RT)	85% (200 cycles)	Wet chemistry, 350 °C 6 h, N_2_
LiNi_0.5_Co_0.2_Mn_0.3_O_2_@YF_3_^[^ ^140^ ^]^	2.5–4.3	141.9 (100 mA g^−1^, RT)	84% (200 cycles)	Wet chemistry, 350 °C 6 h, N_2_
LiNi_0.8_Co_0.15_Al_0.05_O_2_@AlF_3_^[^ ^216^ ^]^	2.7–4.3	≈200 (100 mA g^−1^, 55 °C)	84.7% (100 cycles)	Dry coating process, mixed with nanosized AlF_3_ powder
*x*Li_2_MnO_3_·(1–*x*)LiMO_2_ (Li‐rich layered oxides)	–	–	–	–
Li[Li_0.19_Ni_0.16_Co_0.08_Mn_0.57_]O_2_@AlF_3_^[^ ^142^ ^]^	2.0–4.6	206 (100 mA g^−1^, RT)	79.6% (100 cycles)	Wet chemistry, 400 °C 5 h, N_2_
0.5Li_2_MnO_3_·0.5LiNi_0.5_Co_0.2_Mn_0.3_O_2_@AlF_3_^[^ ^137^ ^]^	2.0–4.6	216.2 (125 mA g^−1^, RT)	98% (50 cycles)	Wet chemistry, 400 °C 5 h, Ar
Li_1.2_Mn_0.54_Ni_0.13_Co_0.13_O_2_@CeF_3_^[^ ^217^ ^]^	2.0–4.6	223 (25 mA g^−1^, RT)	91.7% (50 cycles)	Wet chemistry, 450 °C 2 h, air
Li_1.2_Mn_0.52_Co_0.08_Ni_0.2_O_2_@SmF_3_^[^ ^218^ ^]^	2.0–4.8	165.4 (500 mA g^−1^, RT)	84.5% (150 cycles)	Hydrothermal, 450 °C 2 h, Ar

### 4.1 Wet Chemistry‐Based Coating Route

The wet chemistry‐based methods are extensively used to synthesize designated surface coating layers through chemical reactions in aqueous/nonaqueous solutions. Such treatment routes are facile and scalable for applying in manufacture, which usually follows several steps:^[^ ^26^ ^]^ 1) preparing organic/aqueous precursor solution containing metal salt; 2) immersing the “nuclear” material into the solution and fully dispersed to form a uniform sol system; 3) adding precipitant and changing the solution environment to precipitate the desired “shell” material. However, in most cases, the precipitation rate is too fast to induce a homogenous nucleation, so that a reagent or operation capable of finely tuning the precipitation condition is quite needed to make reactions controllable. Apart from adding slow releasing precipitant,^[^ ^126^, ^127^, ^128^, ^129^ ^]^ one of the most common ways is slow dripping. 4) Evaporating solvents or filtrating solution to separate electrode materials; 5) drying to remove residential solvent followed by postannealing to develop ideal interphase. As a matter of fact, for those most widely studied metal fluoride, their low solubility, which determines faster precipitation behaviors than their oxide or phosphate counterparts, is favorable for heterogeneous nucleation in the form of phase separation or discontinuous “point coating.”^[^ ^130^, ^131^, ^132^, ^133^ ^]^

**Figure 12:**
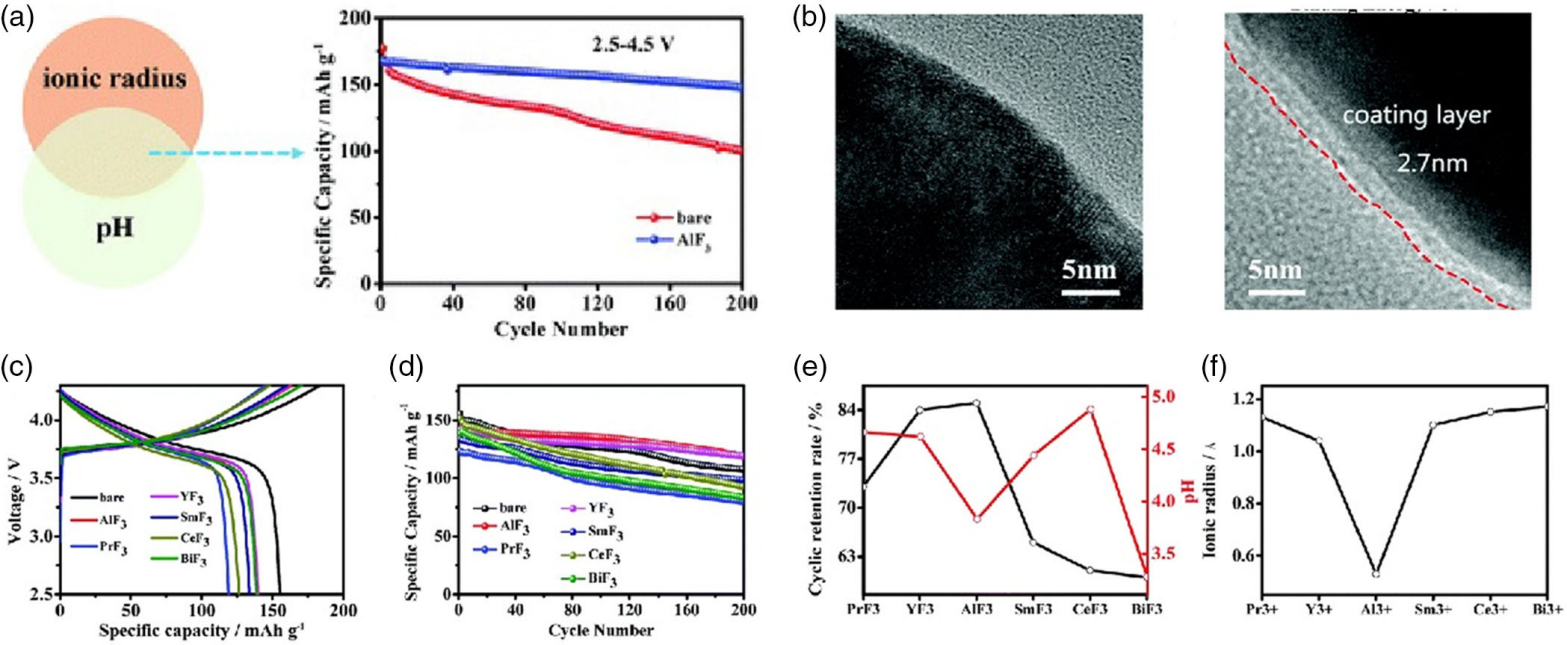
a) The cycling performance of uncoated and AlF_3_‐coated NCM523. The cycling performance was studied at a current density of 100 mA g^−1^. b) HRTEM images of uncoated and AlF_3_‐coated NCM523. c,d) The initial charge/discharge curve and cycling performance of bare, AlF_3_‐, PrF_3_‐, YF_3_‐, SmF_3_‐, CeF_3_‐, and BiF_3_‐coated NCM523 samples at a current density of 100 mA g^−1^ in the voltage range from 2.5 to 4.3 V. e) The pH values and cyclic retention rates and f) ionic radii of the different fluoride coatings. Parts (a‐f): Reproduced with permission.^[^ ^140^ ^]^ Copyright 2020, The Royal Society of Chemistry.

Different fluorides passivation layers such as AlF_3_, CaF_2_, and LiF can be easily constructed by wet chemical method on various types of cathodes. Sun et al.^[^ ^134^ ^]^ first introduced AlF_3_ as a new coating material of LCO to improve its high‐voltage stability at 4.5 V cutoff via direct chemical reaction between Al(NO_3_)_3_·9H_2_O and NH_4_F in water. The molar ratio of F to Al is set as 3 along with slowing adding NH_4_F solution to guarantee a AlF_3_‐coated surface. Consequently, the LCO wrapped up by 2 wt% AlF_3_ showed much improved cycling performance and rate capability with a capacity retention of 85% after 500 cycles in full cell at 4.4 V upper limit in contrast to much deteriorative cycling behavior for pristine LCO. In addition, lower charge transfer resistance and reduced transition metal ions dissolution were also observed in AF_3_‐coated sample, which accounted for improved electrochemical performance. Thereafter, AlF_3_ along with other metal fluorides based on similar synthetic routes is coated on different types of cathodes such as NCM111,^[^ ^135^ ^]^ NCM811,^[^ ^132^ ^]^ LNMO,^[^ ^136^ ^]^ and *x*Li_2_MnO_3_·(1–*x*)LiMO_2_^[^ ^137^ ^]^ to stabilize the interface between the cathode and electrolyte, but many of them show limited protective ability in the form of low crystallized broken particles with rough morphology rather than continuous amorphous phase with higher Li^+^ diffusivity (e.g., 9.26 × 10^−16^ cm^2^ s^−1^ for amorphous AF_3_,^[^ ^138^ ^]^ 6.2 × 10^−18^ cm^2^ s^−1^ for crystalline AF_3_^[^ ^139^ ^]^) because most of the researches on fluoride modification are done via a trial‐and‐error method, lacking in element selectivity and screening criteria. Therefore, Xie et al.^[^ ^140^ ^]^ concluded a general rule that a high‐quality fluoride coating will be achieved with fluoride suspension pH of ≈4.0 and small metal cation ionic radius. According to this principle, AlF_3_ and YF_3_ are proved to be favorable coatings for the structural stability of NCM cathode (**Figure** **12**). In view of comprehensive consideration of phase stability, (electro)chemical stability and Li ion conductivity, several Li‐containing fluorides Li_2_MF_6_ (M = Si, Ge, Zr, Ti) have also been identified to be valid by means of high‐throughput computational screening approach.^[^ ^141^ ^]^

**Figure 13:**
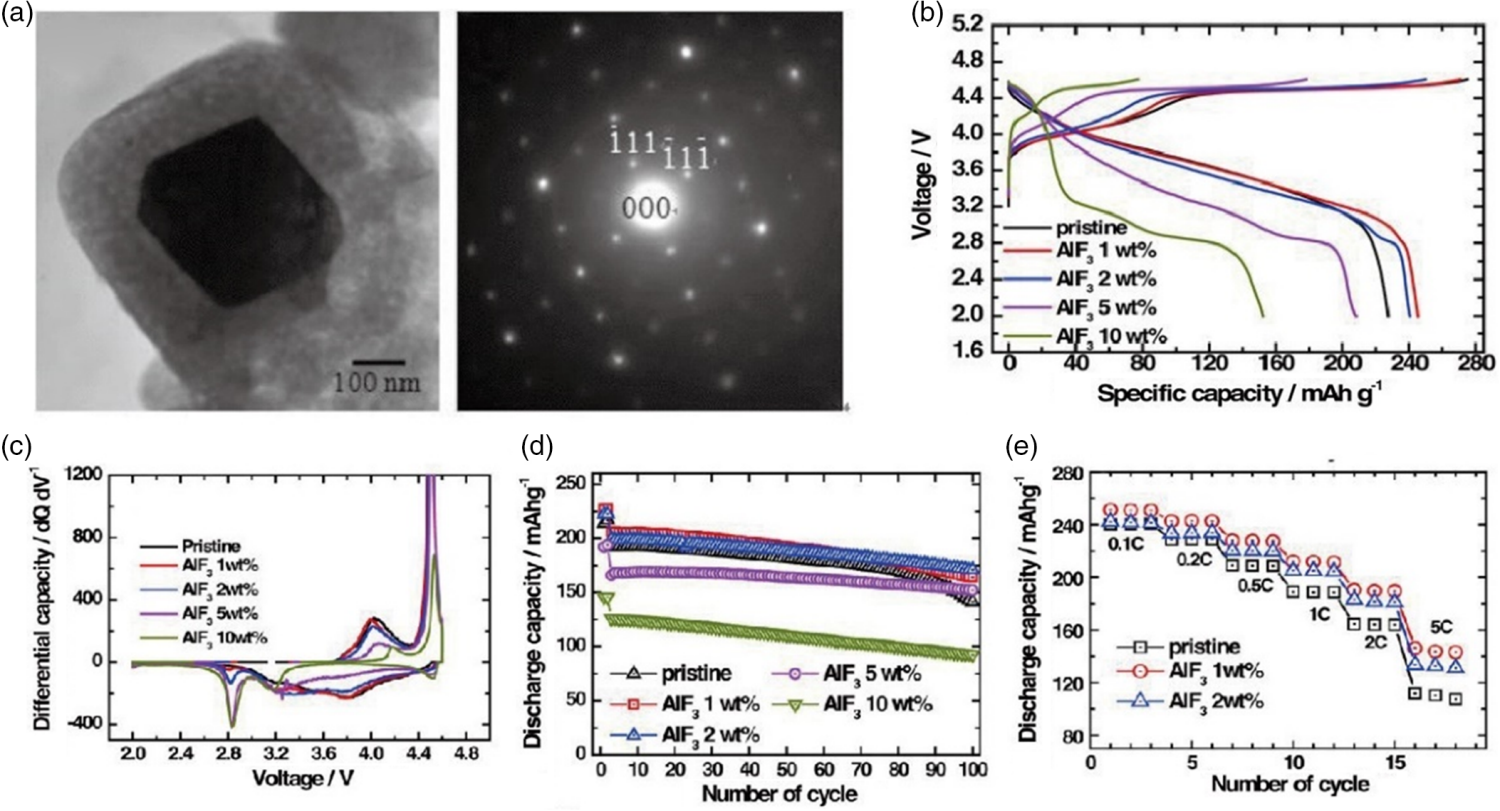
a) Primary particles from 5 wt% AlF_3_‐coated sample that was completely encapsulated by the AlF_3_ coating, and its electron diffraction pattern. b) Initial charge–discharge curves. c) Corresponding dQ dV^−1^ versus V curves of pristine and AlF_3_‐coated electrodes. d) Cycling stability and e) rate capability of pristine and AlF_3_‐coated electrodes. Parts (a‐e): Reproduced with permission.^[^ ^142^ ^]^ Copyright 2012, Wiley‐VCH.

In addition, the performance of cathodes greatly depends on the amount of fluorides coating because overly thick coating will sacrifice initial reversible capacity and degrade charge‐transfer kinetic, leading to elevated interfacial impedance due to its electrochemical inertness and poor conductivity. For example, Sun et al.^[^ ^142^ ^]^ indicated that Li‐rich material, Li[Li_0.19_Ni_0.16_Co_0.08_Mn_0.57_]O_2_, showed the best cyclability of 85.9% with an optimal addition of 2 wt% AlF_3_. Interestingly, a transformation of the initial layered Li_2_MnO_3_ to spinel phase induced by AlF_3_ coating layer has been substantiated to be beneficial to address the intrinsic shortcomings of Li‐rich cathode via electrochemical measurements and TEM analysis, which are featured with discharge plateau at 2.8 V, characteristic oxidation peak of spinel structure at 4.18 V, and typical electron diffraction pattern (**Figure** **13**). This work revealed an extraordinary surface stability mechanism that is completely different from previous cognition brought by fluorides.

Notably, the fluorides modification seems to play a multiple role in interphase stability. Myung^[^ ^143^ ^]^ found that AlF_3_ mitigated the oxygen evolution of delithiated NCM111 at high temperature with less weight loss. As a result, the phase transformation to cubic spinel structure was retarded, implying positive effect of AlF_3_ on thermal behavior. However, the modified electrochemical properties brought by wet chemistry‐based fluorides coating are debatable as NMR spectra show no evidence for the formation of pure AlF_3_ after 400 °C sintering; instead, a partially fluorinated Al_2_O_3_ spinel‐like phase derived from hydrated aluminum oxyfluoride appears as HF‐scavenger to impact the mechanism.^[^ ^144^ ^]^ It is also expected that evolution of bulk structure caused by Li^+^/proton exchange reaction during solution‐based coating process will affect the judgment of results as well, particularly in Ni‐rich systems.

### 4.2 Hydrothermal Coating Routes

Hydrothermal method is a versatile synthetic platform for functional core–shell structure from high‐temperature solutions at high vapor pressure. In a common hydrothermal‐assisted coating process, the reaction precursor including metal source and precipitant is fully dissolved in a specific solvent in advance followed by adding electrode materials to the solution. After stirring to form a homogenous mixture, the solution will be transferred into a sealed Teflon autoclave, and the temperature of hydrothermal keeps at preset value for a period of time to precipitate the target product. At last, filtration, washing, drying, and postsintering procedures are conducted in sequence to achieve final products.^[^ ^145^, ^146^, ^147^, ^148^ ^]^ The coatings prepared by hydrothermal reactions have the advantages of well crystallization and adjustable composition. Meanwhile, the disadvantages are prominent with harsh conditions, invisible growth process, low yield, and lack of nanoprecision.

**Figure 14:**
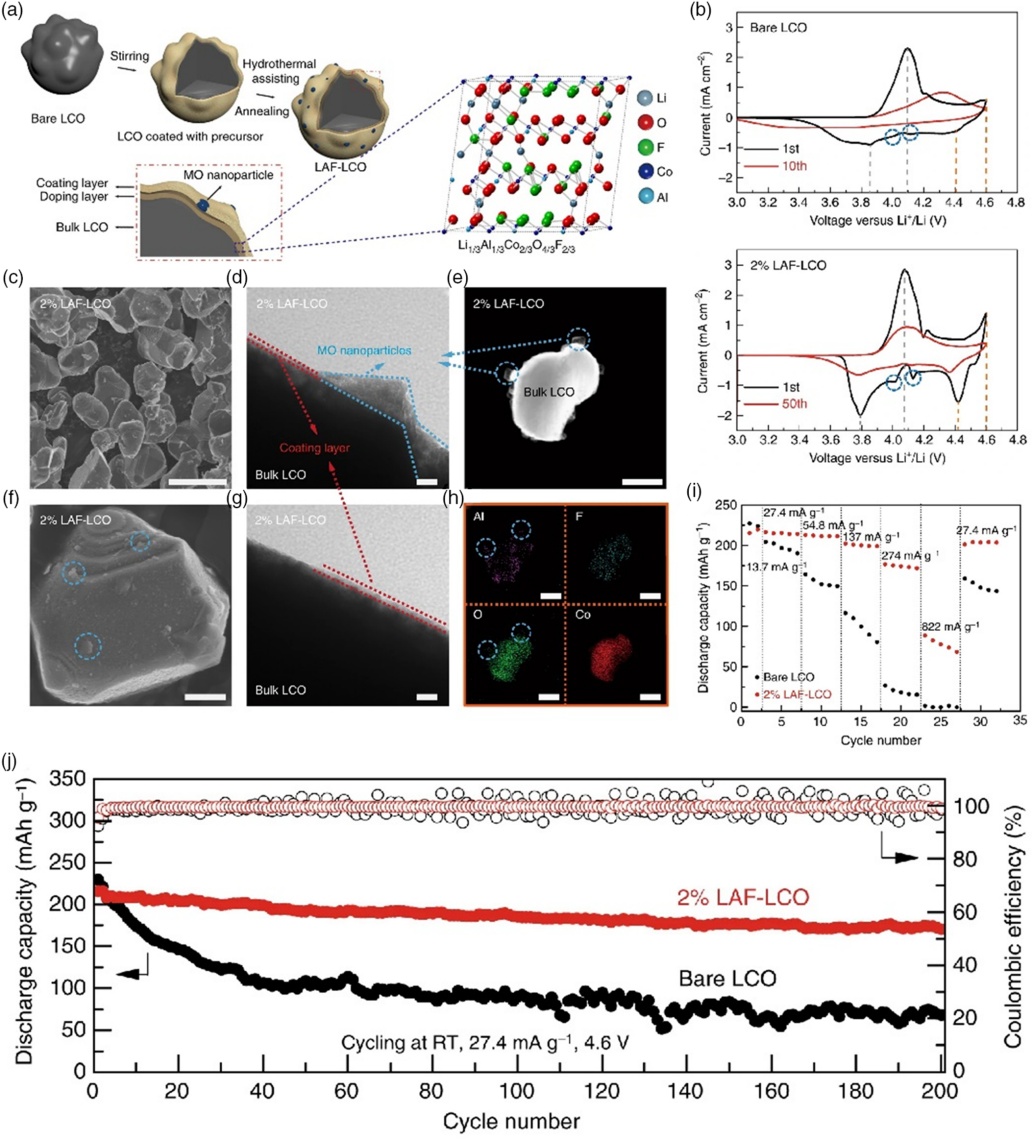
a) The formation and synthetic process for constructing LAF–LCO and the most probable supercell structure of Li–Al–Co–O–F solid solution using stoichiometric calculations. b) Cyclic voltammograms of cells with bare LCO or 2% LAF–LCO electrodes at a scan rate of 0.1 mV s^−1^ in the voltage range of 3.0–4.6 V (vs Li^+^/Li). c‐h) Morphological and structural analyses of LAF–LCO. i) Rate performance of half‐cells with bare LCO and 2% LAF–LCO electrodes at room temperature in the voltage range of 3.0–4.6 V (vs Li^+^/Li). j) Long‐term cycling performance of half‐cells with bare LCO or 2% LAF–LCO electrodes at room temperature in the voltage range of 3.0–4.6 V (vs Li^+^/Li) at current density of 27.4 mA g^−1^. Parts (a‐j): Reproduced under the terms of the CC‐BY 4.0 license.^[^ ^149^ ^]^ Copyright 2018, The Authors, published by Springer Nature.

In addition to metal oxide coatings, the hydrothermal‐assisted method could also prepare metal fluoride with subtle texture of subsurface. Qian et al.^[^ ^149^ ^]^ demonstrated that such a modification strategy could contribute to excellent electrochemical performance of LCO at a high cutoff voltage of 4.6 V. First, a ternary lithium–aluminum–fluorine (LAF) hybrid layer was constructed on the LCO surface and then annealing treatment initiated LAF doping to form a subsurface of Li–Al–Co–O–F solid solution with sparse Al_2_O_3_/LiAlO_2_ nanoparticles (**Figure** **14**a,c‐h). The functional double‐layered structure not only suppressed harmful irreversible phase transition from O3 to H1‐3 but also ensured fast Li^+^ diffusion within coating layer by introduction of excess Li (Figure 14b,i). After that, the structural integrity and interphase stability are both improved to realize excellent cycling performance with a capacity of 170.7 mAh g^−1^ at 27.4 mA g^−1^ and a capacity retention of 81.8% after 200 cycles for 2% LAF‐modified LCO much superior to that of bare sample with only 32.8% (Figure 14j). It is noteworthy that anodic (F) and cationic (Li, Al) codoping was presented as solid solution beneath outermost surface with most likely formula of Li_1/9_Al_1/3_Co_2/3_O_4/3_F_2/3_ predicted by DFT calculations and corroborated by the Al, O, F characteristic peaks of Li–Al–O–F compound in XPS. Such surface hybrid modification from coating to partial doping provides insight into interaction between the surface coating and the crust of the cathode materials, which offers inspiration and guidance for interface design in future.

### 4.3 Atomic Layer Deposition Coating Routes

**Figure 15:**
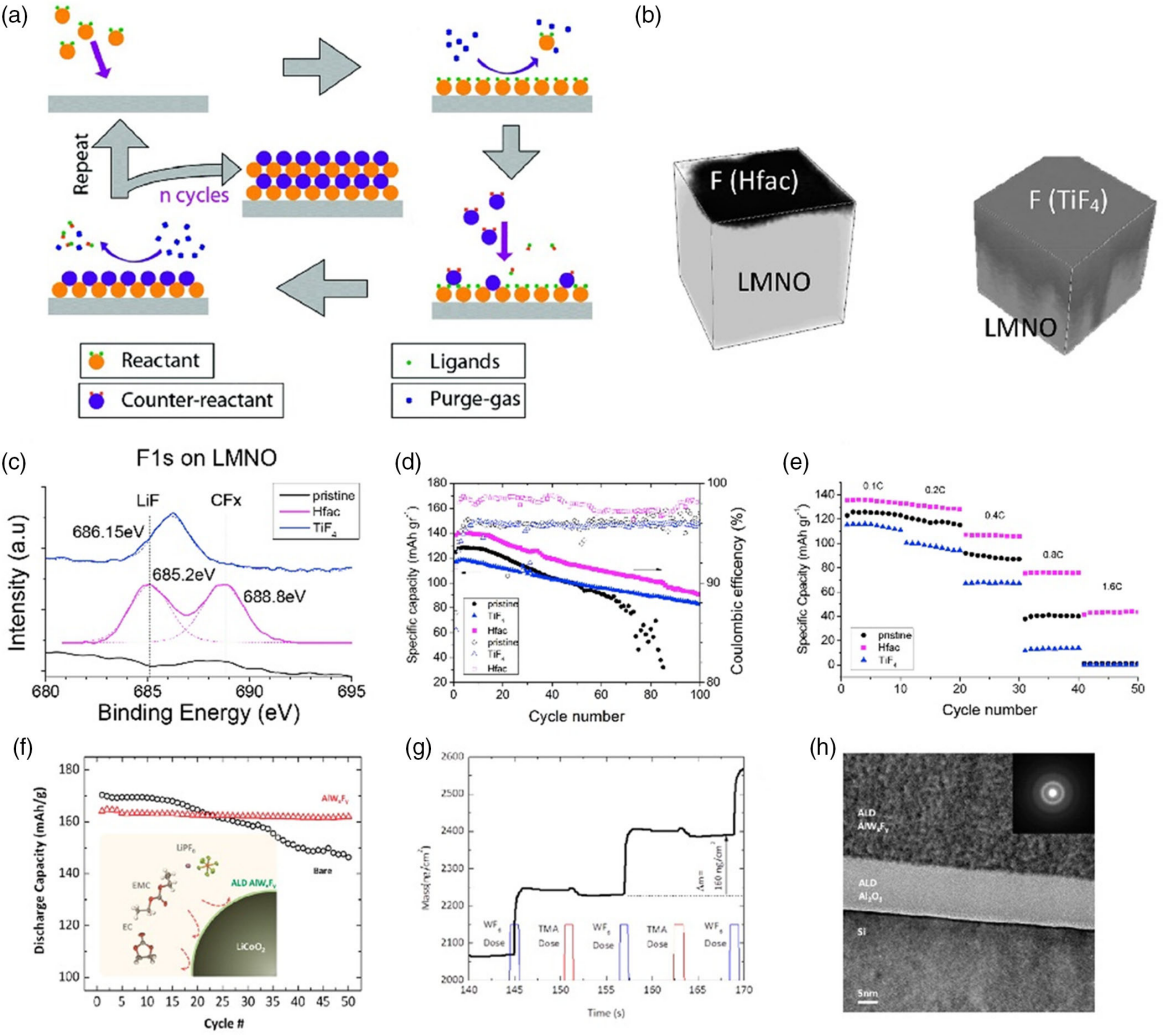
a) Schematic of the sequential ALD process. b) 3D rendering of the surface of F^−^ on LMNO powder for Hfac‐ and TiF_4_‐coated materials from TOF‐SIMS. c) High‐resolution XPS measurements performed on pristine and coated LMNO powder of F1s. Pristine and coated electrodes in 1M LiPF_6_ EC:DMC half‐cells versus Li metal in an operating window of 3.5–4.85 V (0.1 mA cm^−2^) were cycled. d) Discharge capacity and columbic efficiency versus cycle of pristine and coated powder electrodes. e) C‐rate results of cells in a similar configuration. f) Cyclic performances of bare (black) and AlW_*x*_F_*y*_‐coated (red) Li^+^/LiCoO_2_ cells at a current rate of 20 mA g^−1^ between 4.4 and 2.5 V. g) In situ QCM mass uptake during two alternating cycles of WF_6_ and TMA. h) HRTEM image of the films deposited using alternating exposures to TMA‐WF_6_ on an ALD Al_2_O_3_‐coated Si wafer. Part (a): Reproduced with permission.^[^ ^150^ ^]^ Copyright 2015, The Royal Society of Chemistry. Parts (b‐e): Reproduced with permission.^[^ ^154^ ^]^ Copyright 2020, Elsevier. Parts (f‐h): Reproduced with permission.^[^ ^155^ ^]^ Copyright 2015, American Chemical Society.

To handle the challenges of atomic precision film and tunable thickness that cannot be fully satisfied by conventional wet chemical‐based methods, some chemical vapor deposition technology has been used to achieve ultrathin homogenous coating with nanoaccuracy on cathode materials for LIBs. As a branch of chemical vapor deposition, atomic layer deposition (ALD) technology occurs through sequential self‐limiting gas‐phase reactions on the surface of substrate. The surface saturation determines the amount of precursor absorption that means the net absorption rate of precursors approaches to zero at this point, and there will be no extra increase in thickness regardless of surface exposure time.^[^ ^150^, ^151^ ^]^ Therefore, it is the self‐limiting nature that endows ALD with ability to control the coating layer down to 0.1 nm. As illustration depicts in **Figure** **15**a, a whole ALD cycle includes four program steps: 1) a gaseous reagent, usually volatile organometallic compound, is pulsed into the reaction chamber to absorb on the substrate until saturated, thus generating intermediate and byproducts; 2) excess reactants and byproducts are removed by inert gas purging; 3) a counter‐reactant is then pulsed into the system to trigger reaction with the intermediate absorbed and a thin film with a thickness of several atomic layers (≈1 Å) is produced; 4) Excess counter‐reactants are swept away by inert gas blowing again. After repeated cycles, the ALD deposited conformal surface film gets thicker.

In recent years, the ALD techniques have achieved success in preparing coatings of metal oxides,^[^ ^152^ ^]^ phosphates,^[^ ^153^ ^]^ and conductive polymers^[^ ^118^ ^]^ for interphase modification of cathodes. In regard to metal fluorides, ALD method is also applicable but faced with the problem of selecting suitable fluorine precursors. Tiurin et al.^[^ ^154^ ^]^ utilized the ALD technique to construct LiF coating on LNMO cathode with two distinct F source and compared their differences in chemical composition and electrochemical performance. The results showed that electrodes coated with hexafluoroacetylacetone (Hfac)‐derived LiF possessed best long‐term cycling performance, excellent power capabilities along with higher Li content exceeding its TiF_4_‐derived and pristine counterparts (Figure 15d‐e). These differences were ascribed to the formation of conductive hybrid LiF‐CF_*x*_ layer rather than pure LiF when using Hfac as F source, leading to a more porous surface in favor of Li^+^ transport (Figure 15c). When it comes to utilizing TiF_4_ as F source, a more prominent Li diffusion into bulk and Li surface deficiency was observed by TOF‐SIMS (Figure 15b) probably owing to adhesion issues and serious HF etching. Although ESI measurements demonstrated increased CEI resistance, an improved interphase stability could still be achieved in both cases, indicating that even a small amount of LiF contributed to stable CEI.

To further enhance the conductivity of fluoride protective layer, composite polymetallic‐fluoride film can be deposited on electrodes via alternating exposures of metal precursors using ALD (Figure 15g).^[^ ^155^ ^]^ A AlW_*x*_F_*y*_ thin film with thickness of only 1 nm could be produced by five ALD cycles of TMA–N_2_–WF_6_–N_2_ on flake LCO (Figure 15h). Such binary metal fluoride layer combined chemical inertness of AlF_3_ with highly conductivity of metallic tungsten, enabling a capacity retention of 99% after 50 cycles at 20 mA g^−1^ when the cutoff voltage was set to 4.4 V versus Li^+^/Li (Figure 15f). Therefore, ALD makes it possible to design advanced surface structures containing multiple components for next‐generation LIB cathode materials.

## 5 Fluorinated SEI

At present, LMBs are facing serious safety problem related to dendrite growth, the production of “dead Li” and side reactions between Li anode and electrolyte, which limits the application of metallic Li with a much low critical current density. It is well recognized that the initial nucleation sites of Li play a leading role in the dendrite formation process, and surface energy and Li^+^ diffusion barrier are the two most important factors.^[^ ^156^, ^157^, ^158^ ^]^ Therefore, to enhance mechanical strength and reduce diffusion barrier, resulting in improved Li metal interphase stability, many strategies have been made to render uniform Li deposition, including ether‐based electrolytes,^[^ ^159^ ^]^ solid‐state electrolytes,^[^ ^160^ ^]^ Li‐based alloys,^[^ ^161^ ^]^ nanostructured 3D conductive scaffolds,^[^ ^162^ ^]^ and various electrolyte additives. Among these, designing fluorinated SEI, particularly LiF‐enriched interface, gains increasingly popularity in recent years given fluoride's prevalence in almost all native SEIs due to its unique properties. Depending on the high mechanical strength, wide electrochemical stability window (0–6.4 V versus Li^+^/Li)^[^ ^163^ ^]^ and low Li diffusion barrier of LiF, fluorinated SEI is suggested to be beneficial to Li deposition behavior in high‐voltage LMBs and expected to realize prolonged cycling with high coulombic efficiency. Therefore, a comprehensive summary on fabricating fluorinated SEI is quite desirable to make a deep understanding of the source of improved electrochemical performance. Various synthesized strategies including ex situ and in situ approaches have been extensively studied to realize dendrite‐free stable cycling, as shown in **Table** **3**, for further comparison of their eletrochemical differences in Li|Li symmetric cells.

**Table 3:** A comparison of the electrochemical performances of fluorinated interfaces in li|li symmetric cells

Fluorination methods		Electrolyte composition	Cycling stability	Current density [mA cm^−2^]
Ex situ	LiF additives^[^ ^117^ ^]^	1 m LiPF_6_ EC:DMC‐0.5 wt% LiF	Over 140 h	4
Ex situ	NH_4_F treatment^[^ ^39^ ^]^	Ta‐doped Li_6.4_La_3_Zr_1.4_Ta_0.6_O_12_	Over 200 h	0.5
Ex situ	Freon R134a gaseous reaction^[^ ^169^ ^]^	1 m LiPF_6_ 1:1 v v^−1^ EC:DEC	Over 450 h	1
Ex situ	CYTOP thermal decomposition^[^ ^168^ ^]^	1 m LiPF_6_ 1:1 v v^−1^ EC:DEC	Over 120 h	5
Ex situ	LiF ALD on h‐BN^[^ ^170^ ^]^	1 m LiPF_6_ 1:1 v v^−1^ EC:DEC	Over 300 h	0.5
Ex situ	SnF_2_ solution drop‐casting^[^ ^171^ ^]^	1 m LiPF_6_ 1:1 v v^−1^ EC:DEC	Over 800 h	1
Ex situ	CuF_2_‐containig solution reaction^[^ ^172^ ^]^	1.0 m LiTFSI 1:1 v v^−1^ DOL:DME	Over 800 h	2.5
Ex situ	AlF_3_ framework lithiation^[^ ^182^ ^]^	1 m LiPF_6_ EC/DEC‐FEC‐VC	Over 100 cycles	20
In situ	HCE^[^ ^201^ ^]^	10 m LiFSI EC/DMC	1000 h	1
In situ	LHCE^[^ ^203^ ^]^	1.2 m LiFSI/DMC‐BTFE (1:2)	200 cycles	0.5
In situ	LiFSI electrolyte‐soaking^[^ ^209^ ^]^	Li_3_PS_4_ solid‐state electrolyte	350 h	0.3
In situ	Li–Sr alloy electrolyte immersion^[^ ^210^ ^]^	1 m LiPF_6_ FEC:FEMC:HFE	180 cycles	30
In situ	LiPF_6_/NaPF_6_ electrolyte soaking^[^ ^211^ ^]^	1 m LiPF_6_ EC/DEC	3000 h	0.5
In situ	All‐fluorinated electrolyte^[^ ^191^ ^]^	1 m LiPF_6_ in FEC/FEMC/HFE	Over 500 cycles	0.5
In situ	LiTFSI‐LiBOB dual‐salt electrolytes^[^ ^206^ ^]^	LiPF_6_‐LiTFSI‐LiBOB EC/EMC	210 h	1
In situ	Fluorinated orthoformate electrolytes^[^ ^189^ ^]^	1 m LiFSI/DME‐TFEO	Over 700 h	0.5
In situ	Ether‐based LHCE^[^ ^204^ ^]^	LiFSI‐1.2DME‐3TTE	Over 300 cycles	0.5
In situ	DFEC‐based electrolyte^[^ ^188^ ^]^	1.2 m LiPF_6_ DFEC/EMC	1000 h	2

### 5.1 Ex Situ Fluorination Strategies of Artificial SEI

**Figure 16:**
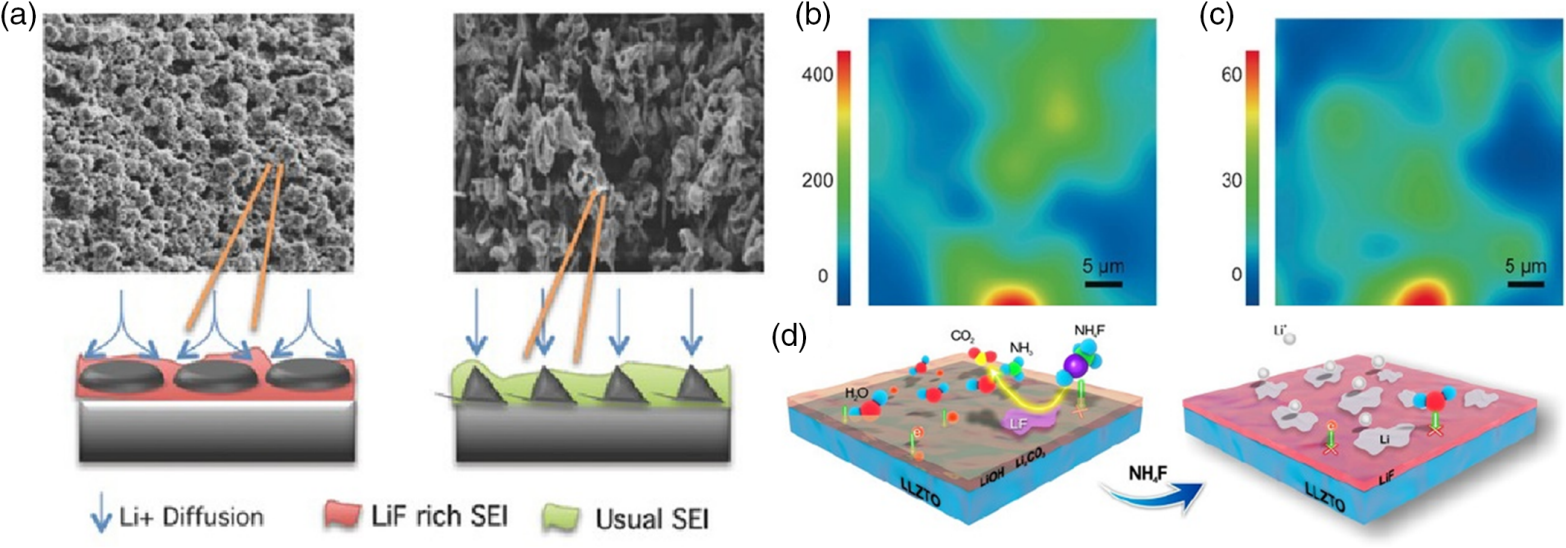
a) Pictorial representation of proposed mechanism: lithium diffusion near the surface of electrodes represented by blue arrows and corresponding surface morphology of lithium anodes polarized at 2 mA cm^−2^ for 2 h. Raman mapping of b) LLZT and c) LLZT‐2LiF after 2 weeks of exposure in air. d) Schematic illustration showing the conversion from residual‐Li‐contaminated LLZTO to LiF‐coated LLZTO by NH_4_F treatment, and the surface stability against air exposure and hostless evolution of Li metal. a) Reproduced with permission.^[^ ^117^ ^]^ Copyright 2016, Wiley‐VCH. Parts (b,c): Reproduced with permission.^[^ ^166^ ^]^ Copyright 2016, Wiley‐VCH. Part (d): Reproduced with permission.^[^ ^39^ ^]^ Copyright 2020, Wiley‐VCH.

LiF is regarded as an effective additive in controlling surface diffusion of Li^+^ during deposition because LiF‐modified surface has lower barrier energy than other SEI components for Li^+^ diffusion, leading to faster rate of Li^+^ transport. In addition, LiF interphase has higher bulk modulus than carbonate substrate (70 GPa for LiF vs 63 Gpa for Li_2_CO_3_)^[^ ^164^ ^]^ that means it has less tendency to be penetrated by lithium dendrite with greater mechanical hardness in favor of a smooth surface. Inspired by the function of LiF in SEI, for the first time, Choudhury^[^ ^117^ ^]^ introduced LiF as a direct additive to conventional carbonate electrolytes, LiPF_6_ EC:DMC. The results showed only 0.5 wt% of LiF in a 1 M electrolyte solution could offer sufficient dendrite inhibition ability and slow down cell degradation during cycling. The LiF additive contributed to a lower interface resistance and higher coulombic efficiency more than 90% when compared with bare electrolyte. In addition, a smooth surface morphology of lithium anode was also observed in symmetric cells for scanning electron microscope (SEM) analysis (**Figure** **16**a). This facile and viable strategy achieved success in LiFePO_4_ half‐cell with much improved cyclability and discharge capacity for 150 cycles, which confirms the validity of LiF additives. Moreover, infusing liquid electrolytes reinforced with halogenated salt blends [1 m (0.7 LiTFSI + 0.3 LiF)‐PC] in the pores of a nanoporous ceramic (alumina/poly(vinylidene fluoride) (PVDF) membrane) caused much extended lifetimes of symmetric lithium cells for over 350 h at a current density of 0.5 mAh cm^−2^.^[^ ^165^ ^]^ Such outstanding room‐temperature LMB performance originated from synergetic effect of LiF additive‐stabilized lithium deposition which was proved by previous JDFT calculations, and high‐conductivity and modulus of ceramic separators. It is fully testified that optimizing electrolyte ingredients by lithium halide salts as additives is an easy‐to‐use strategy for stabilizing solid–liquid interphase between lithium metal and electrolyte.

To solve the problem of unstable surface of garnet‐type solid‐state electrolyte, Li et al.^[^ ^166^ ^]^ added a small amount of LiF powder to garnet Li_6.5_La_3_Zr_1.5_Ta_0.5_O_12_ (LLZT) after primary calcination and then a highly moisture‐resistant surface was acquired with less reactivity through secondary sintering. The Raman spectra and mapping displayed much weaker signals of CO_3_^2−^ in LiF‐treated samples than pristine ones, indicating smaller Li_2_CO_3_ residues (Figure 16b‐c). The LiF on the surface and grain boundaries not only reduced contact of pellet surface with CO_2_ and water in air, but also eliminated Li_2_CO_3_ distributed inside pellets, which promoted increased stability as well as decreased interfacial resistance with Li metal. As a result, the all‐solid‐state Li/CPEO‐LITFSI/LLZT‐2LiF/LiFePO_4_ battery still delivered 120 mAh g^−1^ capacity during 100 cycles at 80 mA cm^−2^ and exhibited a high coulombic efficiency beyond 99.8%. In fact, a LiF‐rich surface in garnet‐type electrolyte can also be in situ converted from Li_2_CO_3_ contaminants through chemical reactions with NH_4_F dissolved in DMSO at moderate temperature, leaving out high‐temperature removal process (Figure 16d).^[^ ^39^ ^]^ This novel conversion chemistry enabled air stable surface and dendrite‐proof Li plating/stripping at a high critical current density of 1.4 mA cm^−2^.

**Figure 17:**
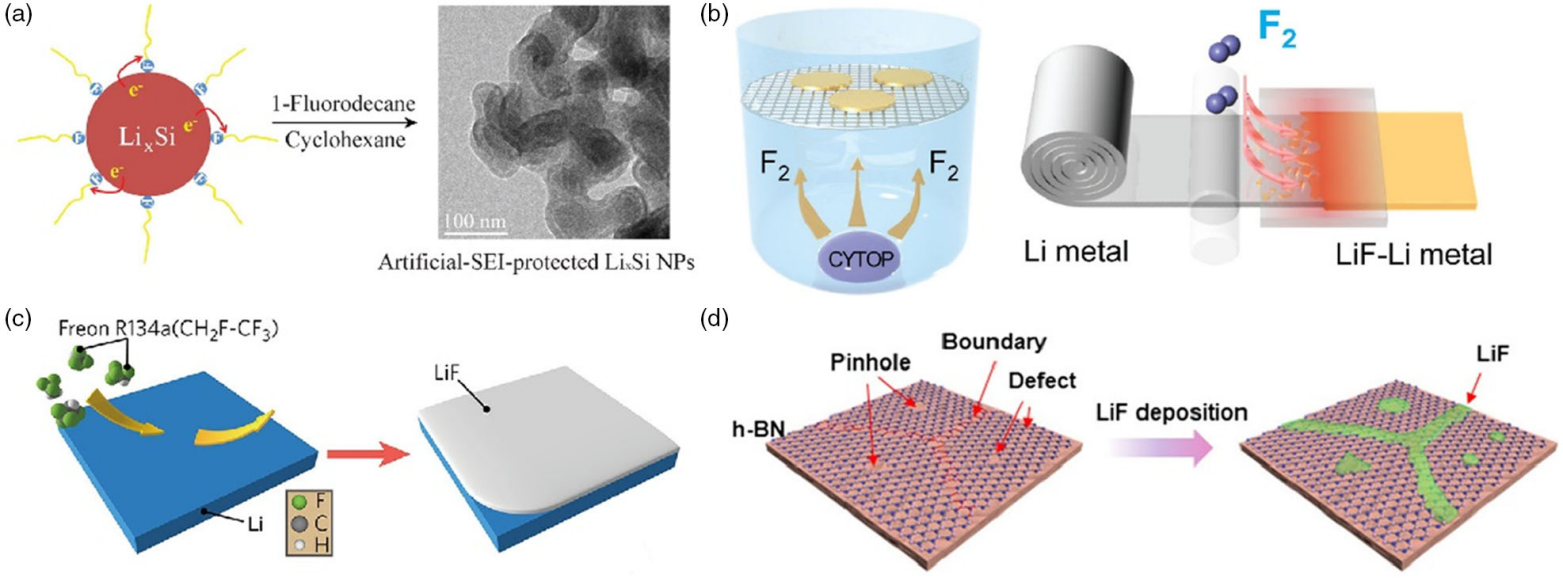
a) Schematic diagram of the artificial SEI coating formed by reduction of 1‐fluorodecane on the surface of Li_*x*_Si NPs in cyclohexane. Reproduced with permission.^[^ ^167^ ^]^ Copyright 2015, American Chemical Society. b) A schematic illustrates that the fluoropolymer, CYTOP, gradually decomposes and releases pure F_2_ gas upon heating, which reacts with Li metal to form a uniform and compact LiF coating. Reproduced with permission.^[^ ^168^ ^]^ Copyright 2017, American Chemical Society. c) Schematic diagrams of surface LiF coating, showing the surface treatment of Li metal with Freon R134a, which helps form a conformal LiF coating on the Li metal surface. Reproduced with permission.^[^ ^169^ ^]^ Copyright 2017, American Chemical Society. d) Schematics of selective ALD LiF deposition on h‐BN. Reproduced with permission.^[^ ^170^ ^]^ Copyright 2017, American Association for the Advancement of Science.

It was found that the fluorination treatment of lithium‐containing anode can also be realized by utilizing the high reactivity of lithium metal and various fluorinating reagents, which could yield a more stable electrode/electrolyte interface free of dendrites with well uniformity. Specifically, Zhao et al.^[^ ^167^ ^]^ invented a facile fluoride coating method to stabilized nanoscale prelithiation reagents Li_*x*_Si, leading to an excellent storage performance in harsh environment (≈10% RH) compatible with large‐scale battery fabrication. As shown in **Figure** **17**a, owing to the reactivity of Li_*x*_Si nanoparticles (NPs), it reduces 1‐fluorodecane on the surface to generate a dense conformal coating, consisting of LiF and lithium alkyl carbonate with long hydrophobic carbon chains, which is similar to the SEI formation in anode. The detailed reaction mechanism is shown as follows

(1) $${F‐({\text{CH}}_{2})}_{9}{\text{CH}}_{3}\overset{{\text{Li}}_{x}\text{Si}}{\to }{\text{ LiF}+\text{Li}‐({\text{CH}}_{2})}_{9}{\text{CH}}_{3}$$

(2) $$\text{Li}‐{\left({\text{CH}}_{2}\right)}_{9}{\text{CH}}_{3}\overset{O_{2}{\text{/CO}}_{2}}{\to }\text{Li}‐\text{OC}(=O){{O‐(\text{CH}}_{2})}_{9}{\text{CH}}_{3}$$

Thereby, these artificial fluorinated SEI‐passivated Li_*x*_Si NPs could dramatically alleviate the initial irreversible capacity loss and exhibit a high capacity of 1600 mAh g^−1^ even in humid air for 6 h.

To further improve the cycle life of lithium metal anode under high current density (>1 mA cm^−2^), a compact pure LiF layer without organic components is urgently pursed to overcome the adverse effects of porous SEI with sparse crystallized LiF domains against electrolyte corrosion. To this end, a nontoxic fluoropolymers CYTOP has been developed as a convenient surface fluorination reagent to passivate Li metal (Figure 17b).^[^ ^168^ ^]^ The formation of a homogenous and compact crystallized LiF rooted in the unique nature of CYTOP that is able to decompose and gradually release fluorine gas at a temperature lower than 250 °C, superior to other F‐containing counterpart such as Teflon and PVDF in view of reaction condition and passivation effect. Owing to the anticorrosion and dendrite‐suppression brought by LiF coating, Li metal anodes exhibited stable cycling over 300 cycles at current densities of 1 and 5 mA cm^−2^. Likewise, commercial Freon R134a (CH_2_F‐CF_3_)^[^ ^169^ ^]^ was also exploited as fluorinated reagents to construct a LiF coating on Li surface through gas/solid reaction conducted in a heated hermitic vessel, which got the edges of nontoxicity, controllable reactivity, and better permeability (Figure 17c). By applying conformal LiF coating onto layered Li‐rGO electrodes in this way, the symmetric cell cycling exhibited improved stability without overpotential growth for over 200 cycles.

However, it is well known that compared with chemical reaction‐based methods, ALD technology has unique advantages for site‐selective lithium fluoride deposition with precise control and by means of that, Xie et al.^[^ ^170^ ^]^ reported LiF deposited on line and point defects of CVD h‐BN using LiOtBu and TiF_4_ as precursors for stable Li‐metal cycling (Figure 17d). The ALD‐based fluorination methods opened up another pathway for feasibility of hybrid LiF/2D materials film applied in LMBs.

**Figure 18:**
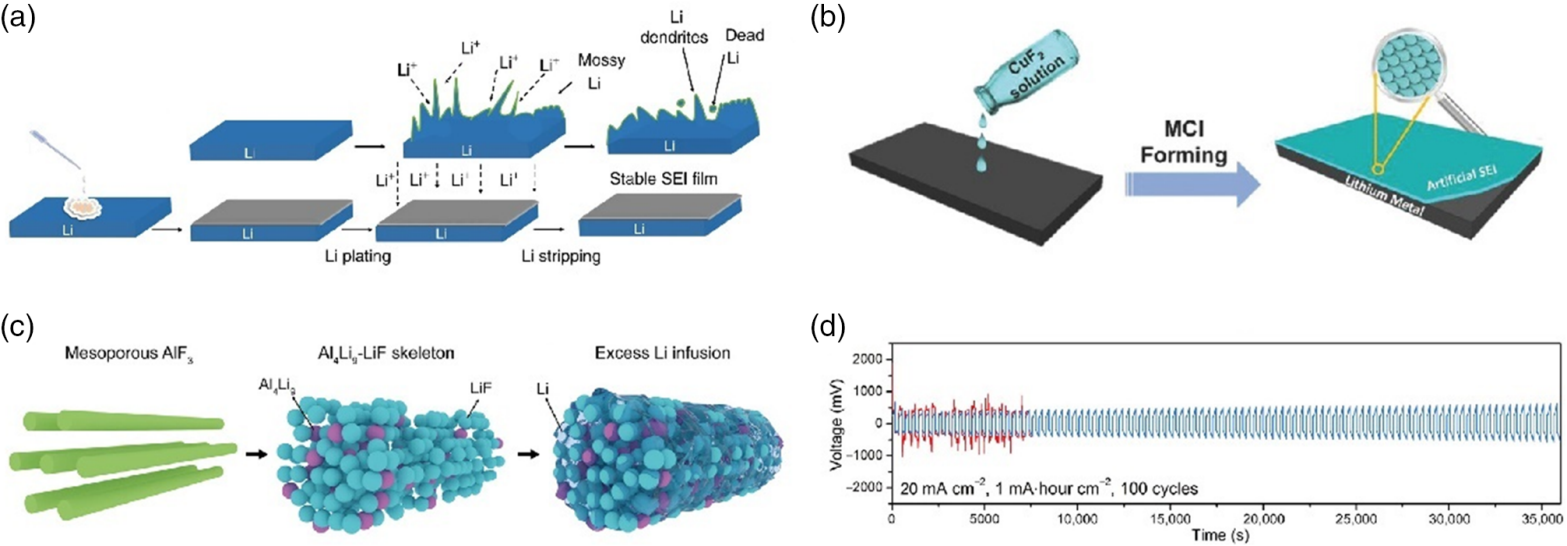
a) Schematic illustration of Li dendrites growth on bare Li and smooth Li deposition on artificial SEI‐protected Li, which is treated with metal fluoride solution. b) A schematic representation of the armored MCI formation. c) Sketch of AlF_3_ powder mesoporous framework, sketch of Al_4_Li_9_‐LiF skeleton, and sketch of Li/Al_4_Li_9_‐LiF (LAFN). d) Symmetric cell cycling performance comparison between Li foil (red) and LAFN (blue) under 20 mA cm^−2^. Areal capacity was fixed at 1 mAh cm^−2^. Part (a): Reproduced under the terms of the CC‐BY 4.0 license.^[^ ^171^ ^]^ Copyright 2020, The Authors, published by Springer Nature. Part (b): Reproduced with permission.^[^ ^172^ ^]^ Copyright 2018, Wiley‐VCH. Parts (c,d): Reproduced with permission.^[^ ^182^ ^]^ Copyright 2017, American Association for the Advancement of Science.

Apart from fluorination reactions that are triggered by gaseous fluorinated reagents on the surface of Li metal, the alloying reactions between metal fluorides and metal lithium combine the advantages of active M‐Li (M=Sn,^[^ ^171^ ^]^ Cu,^[^ ^172^ ^]^ In,^[^ ^173^ ^]^ Zn,^[^ ^174^ ^]^ Bi,^[^ ^175^ ^]^ Au,^[^ ^176^ ^]^ Si,^[^ ^177^ ^]^ Ag,^[^ ^178^ ^]^ etc.) alloy phase with inert but protective LiF to synergic reduce the Li^+^ diffusion barrier and achieve a stable artificial SEI tightly anchored to the Li surface. A replacement reaction occurs between Li metal and MF_*x*_ dissolved in solution (${\text{MF}}_{x}+\;x\text{Li }\to \;x\text{Li }+\text{ M}$) and a uniform LiF/M/M‐Li hybrid layer is generated on the Li metal surface (**Figure** **18**a).^[^ ^171^ ^]^ Typically, an artificial SEI composed of LiF, Sn, and Li–Sn alloy was prepared by drop‐casting electrolyte solutions containing dissolved SnF_2_ onto Li anode, restraining Li dendrite propagation and side reactions. As a result, Li anodes treated by SnF_2_‐containing electrolyte exhibited excellent plating/stripping stability in Li/Li symmetric cells (≈2325 h) at a current density of 0.5 mA cm^−2^, and remarkable cyclability with high‐capacity retention of 80.01% when coupled with NCM111 cathodes. Furthermore, considering the poor ionic conductivity of artificial LiF‐rich SEI on Li metal surface, Cu was investigated as surface modifier and dopant to improve ion diffusion and lithium storage at grain boundaries as well as ensure high surface energy by ex situ forming a composite mixed ionic/electronic conductor interphase (MCI).^[^ ^172^ ^]^ Specifically, dimethyl ether (DME) solution containing LiNO_3_ and CuF_2_ was used as precursor to substitute Li metal and form an MCI film consisting of LiF, Cu, Li_3_N, and LiN_*x*_O_*y*_ with a high Young's modulus of 12.9 GPa, in which Cu atoms were distributed as nanocrystal domains to afford sufficient Li^+^ diffusion pathway (Figure 18b). Such mosaic structure along with dense morphology contributed to stable Li^+^ plating behavior with dendrite‐free formation over 830 h cycling at a large current density of 2.5 mA cm^−2^ and a much‐enhanced interphase stability with smaller diffusion impedance than bare SEI after 300 h cycling.

Structural engineering, particularly the construction of 3D host for Li metal, has been considered as an efficient route to achieve stable plating/stripping in LMBs due to their excellent structural advantage of mixed conductive framework by integrating the electronic and ionic channels together, while for traditional 2D lithium foils, the transport pathways for electrons and ions are separate with each other because the diffusion of Li^+^ occurs on the side of foil near the electrolyte and electrons transfer at the side close to current collector, which makes uniform Li plating impossible.^[^ ^179^ ^]^ In addition, 3D porous structure of host is capable of not only accommodating the volume change in negative electrodes, but also making better interface contact with liquid electrolyte, realizing interconnected mixed conductive channels.^[^ ^180^, ^181^ ^]^ Therefore, many literatures have confirmed the importance and validity of structural design of lithium anode and it includes fluoride frames as well. For example, mesoporous AlF_3_ arrays showed proper spacing for embedding Li metal and great tolerance for volume change. Through an initial overlithiation process of AlF_3_ framework, composite Li/Al_4_Li_9_‐LiF (LAFN) nanoparticles were fabricated and served as ideal reservoirs for Li metal, resulting in excellent dendrite inhibition ability derived from insulating LiF protected Al_4_Li_9_ (Figure 18c).^[^ ^182^ ^]^ Benefiting from this, the symmetric composite anodes delivered an unprecedented high cycling stability over 100 cycles under ultrahigh current density of 20 mA cm^−2^ in contrast to the phenomenon of quick shortcut in common Li foils, indicating its great potential as fast charging/discharging anode for next‐generation LMBs (Figure 18d).

### 5.2 in Situ Fluorination Strategies of SEI

The failure of ex situ artificial F‐enriched SEI is always ineluctable due to the mechanical instability against long‐term plating without remedy to impaired surface, especially in cycling incipient stage, making it intrinsically unstable to protect fresh exposed surfaces of Li metal.^[^ ^183^ ^]^ The construction of ex situ SEI also suffers from relatively fussy procedures alongside fine‐regulated reaction conditions, which plagues its large‐scale application and commercialization prospect. Therefore, it is imperative to develop a functional elastic SEI with fast self‐repairing ability by in situ electrochemical method from the perspective of electrolyte.

#### 5.2.1 F‐Containing Solvents and Electrolyte Additives

Typically, the dominant solvent components located in Li^+^ solvent sheath dictate the decomposition degree of electrolyte and nature of the SEI within multisolvent electrolyte.^[^ ^184^ ^]^ Taking the unique strengths of fluorides into consideration, the F‐containing electrolyte solvent/additive is the first choice to realize efficient and stable Li cycling as it will be preferential reduced by Li anode to generate a large amount of LiF to regulate the interface structure due to its lower LUMO energy level and strong electronegativity of fluorinated groups compared with routine organic solvents.^[^ ^38^ ^]^ Ideally, even if the in situ formed SEI breaks down during repeated plating/stripping, a compact layer of LiF and a flexible organic outer layer will soon be reproduced by electrochemical reduction of F‐containing additives to quickly repair surface film. For this reason, several additive‐based strategies have been proposed to build fluorinated SEI to protect Li metal anode.

**Figure 19:**
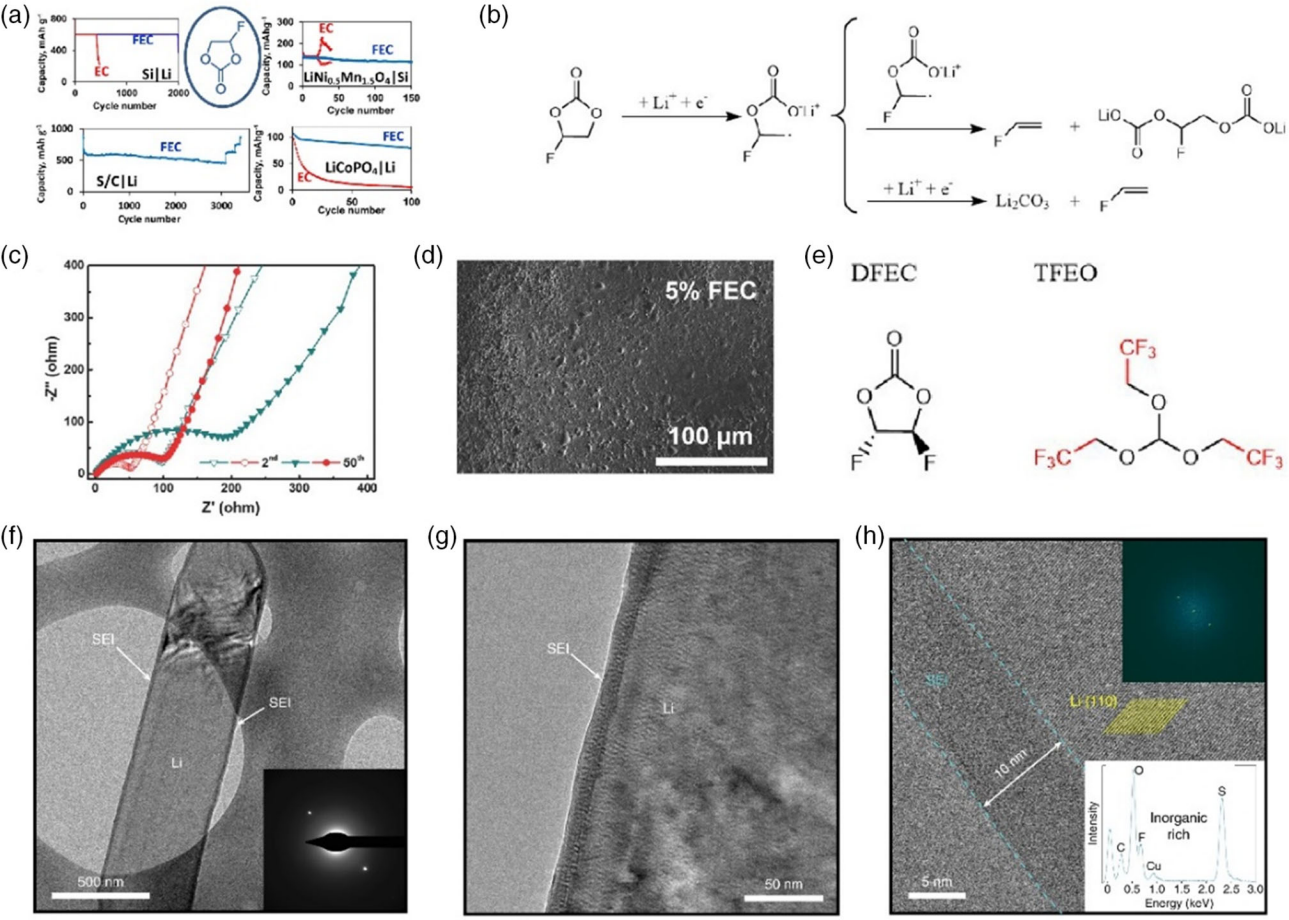
a) Effect of FEC on the cycling performance of Si|Li, LiNi_0.5_Mn_1.5_O_4_|Si, S/C|Li, and LiCoPO_4_|Li cells. b) Possible surface reactions of FEC on Si electrodes to generate CHFCH_2_ intermediate. c) EIS of Li|Cu cells at frequency ranging from 10^5^ to 10^−1^ Hz under amplitude of 10 mV. d) SEM images of Li depositing morphology on Cu foils after 50 cycles with 5% FEC. e) Structural formulas of two additive compounds (DFEC and TFEO). f‐h) Cryo‐EM images of Li deposited on a TEM grid at different scales. Part (a): Reproduced with permission.^[^ ^185^ ^]^ Copyright 2017, American Chemical Society. Part (b): Reproduced with permission.^[^ ^186^ ^]^ Copyright 2013, IOP Publishing. Parts (c,d): Reproduced with permission.^[^ ^187^ ^]^ Copyright 2017, Wiley‐VCH. Parts (f‐h): Reproduced with permission.^[^ ^189^ ^]^ Copyright 2019, Springer Nature.

FEC is one of the most commonly investigated fluorinated additives to alter surface structure on Li metal anode and achieve enhanced coulombic efficiency and capacity retention. To ensure the benefits of FEC over EC‐based electrolytes, Markevich et al.^[^ ^185^ ^]^ compared their performance differences in high‐capacity anodes and high‐voltage cathodes systems, including Si, LiNi_0.5_Mn_1.5_O_4_, LiCoPO_4_, and Li–S. It has been shown that FEC‐based electrolytes manifested outstanding cycling stability in all research systems due to improved protective properties of surface film formed on both positive and negative electrodes (**Figure** **19**a). The predominant LiF over other F‐containing species and high content of oxygen‐free polymers suggested that the reduction of FEC at anode underwent a decarboxylation and defluorination process, which may produce CHFCH_2_ as a reaction intermediate with CO_2_ release, resulting in a layered fluorinated elastic film (Figure 19b).^[^ ^186^ ^]^ In addition to the role of film forming, FEC was believed to suppress the decomposition of electrolyte at cathodes, especially in the case of high voltage (>5 V), owing to its high oxidation stable window. Furthermore, when FEC was used as additives in a small amount (5%) to commercial carbonate‐based electrolyte (1 m LiPF_6_ in EC/DEC (1:1 by volume)), significant performance changes were also observed for high energy density Li|LiNi_0.5_Co_0.2_Mn_0.3_O_2_ full cells with a high initial capacity of 154 mAh g^−1^ at a current density of 180.0 mA g^−1^ and much restrained capacity drop after 100 cycles.^[^ ^187^ ^]^ Meanwhile, Li|Cu equipped with electrolyte containing FEC additives showed largely reduced resistance and smooth Li deposits (Figure 19c‐d), demonstrating the superiority of LiF‐rich SEI derived from FEC for LMBs.

Recently, various cyclic carbonates substituted by fluorine or F‐containing groups have been comprehensively studied to improve the stability of Li anode.^[^ ^188^ ^]^ However, few of them could be used as an effective SEI enabler, indicating that the position of fluorine and molecular structure play a leading role. Among them, trans‐difluoroethylene carbonate (DFEC, formulated in Figure 19e) exhibited more impressive passivating capability than FEC to obtain better performance of Li|NCM622 cell with ultrahigh CE of 99.95%. The lower LUMO energy and higher reduction potential of DFEC determine that it is more easily reduced to form dense fluorinated SEI with higher LiF content. Attributed to this property, it showed smaller potential polarization in Li|Li symmetry cells than FEC‐based electrolyte. Nevertheless, the fluorinated SEI widely reported in previous works is mosaic‐type or multilayered‐type with inhomogeneous morphology insufficient to uniform Li plating/stripping, leading to depletion of electrolytes.

In addition to fluorinated cyclic carbonate FEC and DFEC, novel fluorinated orthoformate solvent, tris(2,2,2‐trifluoroethyl) orthoformate (TFEO, formulated in Figure 19e) have been used to minimize the Li pulverization by forming an amorphous monolithic SEI enriched with inorganic species (Figure 19f‐h).^[^ ^189^ ^]^ The CEI formed on NCM811 was featured with crystalline and LiF‐rich. As a result, Li|NCM811 exhibited fast charging/discharging capability at rates of up to 4 C (≈6 mA cm^−2^) while no dendritic Li formation and detrimental phase evolution were found on anode and cathode, respectively.

**Figure 20:**
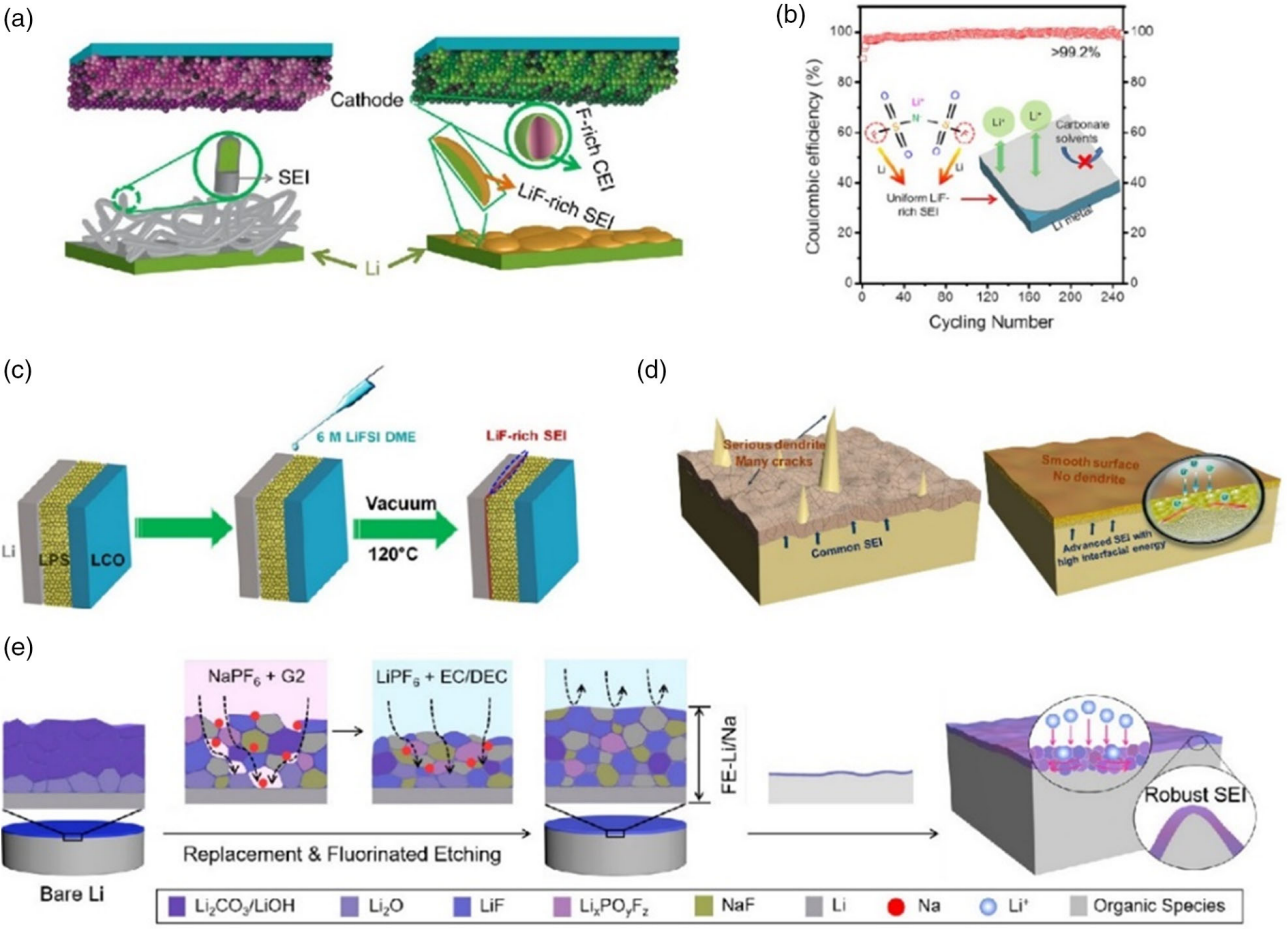
a) Variation of SEI and CEI chemistries formed in different electrolytes: traditional carbonate (left) and all‐fluorinated (right). Reproduced with permission.^[^ ^191^ ^]^ Copyright 2018, Springer Nature. b) Schematic illustration of high reactive fluorine content in the concentrated carbonate electrolyte on a Li‐metal anode and CE of Li deposition/stripping in 10 m LiFSI‐EC/DMC at a current of 0.2 mA cm^−2^. Reproduced with permission.^[^ ^201^ ^]^ Copyright 2017, Elsevier. c) Schematic illustration of the pretreated processes for the formation of an LiF‐rich SEI layer between the Li metal and the LPS solid‐state electrolytes (SSEs). Reproduced with permission.^[^ ^209^ ^]^ Copyright 2018, American Association for the Advancement of Science. d) Schematic diagram comparison of serious dendrite formation in the conventional Li metal anode with unprotected layer (left) and enhanced Li mobility along the interphase and uniform Li deposition in an advanced Li metal anode with high interfacial energy (right). Reproduced with permission.^[^ ^210^ ^]^ Copyright 2020, American Chemical Society. e) Schematic illustration of dendrite suppression by constructing robust SEI on a FE‐Li/Na anode prepared by soaking Li metal first in NaPF_6_ + G2 electrolyte and subsequently in LiPF_6_ + EC/DEC electrolyte. Reproduced with permission.^[^ ^211^ ^]^ Copyright 2021, American Chemical Society.

Considering the broad electrochemical stability window of fluorinated solvents, the concept of all‐fluorinated electrolyte was proposed to achieve excellent safety at aggressive conditions by constructing highly fluorinated CEI/SEI at both cathode and anode simultaneously.^[^ ^190^ ^]^ For example, Fan et al.^[^ ^191^ ^]^ reported a nonflammable all‐fluorinated electrolyte consisted of fluorinated cyclic carbonate, fluorinated acyclic carbonate, and fluorinated alkyl ether and it was utilized for 5 V high‐voltage cathode LiCoPO_4_ and popular high energy cathode NCM811 in LMB. As a result, excellent efficiencies and capacity retention were achieved in all battery systems mentioned earlier owing to the in situ formation of a several‐nanometer fluorinated interphase, which not only suppressed the dissolution of transition metals on the positive side but also enabled dendrite‐free Li anode without increasing the interfacial impedance (**Figure** **20**a). Accordingly, a multifunctional SEI composed of LiF and other inorganic/organic components can be well established by rational designing electrolyte solvents in Li^+^ solvation sheath to obtain desirable features.

#### 5.2.2 F‐Containing Anions

Apart from solvents, it is well recognized that anions in Li salts can also participate in the solvation sheath of Li^+^ and influence the structure of SEI significantly,^[^ ^192^, ^193^ ^]^ while the F‐containing anions such as LiPF_6_, LiBF_4_, lithiumdifluoro(oxalato)borate (LiDFOB),^[^ ^194^ ^]^ lithium bis(fluorosulfonyl) imide (Li[N(SO_2_F)_2_], LiFSI), lithium bis(trifluoromethylsulphonyl)imide (Li[N(SO_2_CF_3_)_2_], LiTFSI),^[^ ^195^ ^]^ lithium (tri‐ﬂuoromethanesulfonyl)(n‐nonaﬂuorobutanesulfonyl)imide (Li[(CF_3_SO_2_)(*n*‐C_4_F_9_SO_2_)N], LiTNFSI),^[^ ^196^ ^]^ and CsPF_6_^[^ ^197^ ^]^ are no exceptions that contribute to fluorinated interphase by donating fluorine, particularly in high salt‐concentrated electrolyte (HCE).^[^ ^198^ ^]^ Unlike free anions in conventional dilute electrolyte, concentrated electrolytes ensure confined solvent and make it possible to recruit more anions in solvation sheath of Li^+^, forming aggregates and ion pairs in solution. The new solution structure transforms the location of LUMO from solvents toward salts and makes the reductive decomposition of salts prior to solvents at a low potential.^[^ ^199^ ^]^ Such design of high concentrated electrolyte guarantees expended electrochemical stability window and flame retardancy with the formation of stable anions‐derived interphase between electrodes and electrolyte.^[^ ^200^ ^]^ Based on the HCE strategy, increasing LiFSI in EC/DMC to 10 m enabled excellent cycling performance of Li | NCM622 battery even at high cutoff voltage of 4.6 V with a capacity retention of 86% after 100 cycles along with CE beyond 99.6%.^[^ ^201^ ^]^ In addition, a more obvious effect on inhibiting lithium dendrite arose from LiF‐rich SEI produced by HCE as evidenced by high Li plating/stripping CE of 99.3% (Figure 20b).

However, the HCE is faced with the challenges of high viscosity and high cost, hampering its practical application. An extra inert solvent is required to dilute the concentrated electrolyte. This diluent should have wide electrochemical window, low viscosity, low cost, and mutual solubility with HCE and cannot dissolve any lithium salts.^[^ ^38^, ^199^ ^]^ Therefore, a localized high‐concentration electrolyte (LHCE) will be achieved with exceptional wettability and conductivity while preserving the local coordination environment of HCE. In the system of LHCE, the strong interactions between salts and solvents within inner Li^+^ solvation sheath contribute to charge transfer among molecules and directly determine the properties of SEI.^[^ ^202^ ^]^ For example, bis(2,2,2‐trifluoroethyl) ether (BTFE) was used as an ideal additive to dilute the concentrated 5.5 m LiFSI/DMC electrolyte, forming a LHCE with saturated LiFSI concentration of 1.2 m.^[^ ^203^ ^]^ The Bader charge analysis showed that BTFE had the weakest interaction with Li anode than the other electrolyte components like DMC or LiFSI, suggesting its inertness. Similar to the case of HCE, FSI^−^ anions possessed the lowest LUMO energy in LHCE that means it will decompose first to form a LiF/Li_2_O rich SEI, as evidenced by XPS. As a result, LHCE enabled dendrite‐free cycling of Li anode and outstanding capacity retention of >80% after 700 cycles for Li|LiNi_1/3_Mn_1/3_Co_1/3_O_2_ when charging at 1 mA cm^−2^ and discharging at 4 mA cm^−2^. It is worth noting that this concept of LHCE can also be applied to other electrolytes such as carbonate‐based ones and ether‐based ones to improve their behavior toward Li metal electrodes and high‐voltage cathodes.^[^ ^204^ ^]^

Furthermore, to improve the Li metal cycling efficiency at large current density along with charging capability, composite salts/dual salts with good compatibility and high ionic conductivity are conceived to be favorable to match metal Li, overcoming the inherent defects of conventional single lithium salt‐like corrosion of Al current collector by LiTFSI. Various robust and conductive fluorinated SEI have been achieved through composite lithium salts, including LiTFSI‐LiBOB^[^ ^205^ ^]^ (0.05 m LiPF_6_ as an additive^[^ ^206^ ^]^), LiTFSI‐LiFSI,^[^ ^207^ ^]^ and LiDFP‐LiBOB/LiFSI/LiTFSI^[^ ^208^ ^]^ to cope with the dendrite growth and low cycle efficiency simultaneously.

The in situ formation of fluorinated interphase through electrolyte conversion reaction with Li metal is a simple and effective general strategy for all‐solid‐state to build a functional interlayer with low electronic conductivity and high interface energy, which allows high critical current density and suppresses Li dendrite growth. Fan et al.^[^ ^209^ ^]^ designed a LiF‐rich SEI between Li_3_PS_4_ (LPS) solid‐state electrolyte and metal Li anode by dropping highly concentrated 6 m LiFSI dimethoxyethane (DME) solution and following drying treatment (Figure 20c). The fast reaction between LiFSI and Li metal at elevated temperature rendered a conform Li‐rich SEI, which blocked the interfacial side reactions, with excellent ability of Li dendrite suppression. In addition, electrochemical stable LiF broadened the window of LPS allowing large current charge and discharge (>2 mA cm^−2^) together with a high coulombic efficiency of 98%.

To further evaluate the lithium dendrite suppression ability of different components, a critical dendrite length *L*_c_ was proposed as criterion to measure the difficulty of dendrite penetration^[^ ^209^ ^]^

(3) $$L_{c}=\frac{2\gamma E}{\pi \sigma^{2}}$$

where *γ* represents the interfacial energy required to form a new Li/SEI interface per unit surface area. *σ* is the stress at the tip of the crack or grain boundary which is mainly determined by the external current. *E* is the bulk modulus. The longer *L*_c_ indicates more excellent dendrite inhibition ability. Therefore, for a given current density, the interfacial dendrite growth has closely negative correlation with the interfacial energy and mechanical strength (Figure 20d).

On the basis of this criterion and DFT, various metal fluorides have been screened and compared with each other to guide a reasonable interphase design. Finally, Liu et al.^[^ ^210^ ^]^ identified that SrF_2_ had higher a *γE* value than LiF so that a SrF_2_‐rich SEI was designed through in situ reaction of a Li–11 wt% Sr alloy in fluorinated electrolyte (2 M LiFSI‐DME). The symmetric Li–Sr/Li–Sr cells showed outstanding cycling stability after 180 cycles at an ultrahigh current density of 30 mA cm^−2^ distinct to bare Li anode which quickly failed within 30 cycles, and the dense deposited lithium beneath SrF_2_‐rich layer confirmed that this electroless electrolyte‐soaking fluorinated strategy was effective in suppressing Li dendrite evolution. More recently, by this way, a fluorinated lithium/sodium hybrid SEI has been constructed by immersing lithium sheet into electrolytes containing sodium salt and lithium salt in sequence (Figure 20e).^[^ ^211^ ^]^ The composite NaF/LiF‐modified SEI possessed both flexibility and high Li^+^ conductivity. Remarkably, it exhibited optimal performances of symmetric cells and full cells when compared with sole LiF or NaF‐modified samples.

Despite the great progress and success have been achieved by fluorinated interphase engineering for LMBs regardless of ex situ or in situ approaches, insufficient critical current density, low area capacity, and narrow operating temperature range are still the tough challenges that impede their application on rechargeable batteries systems due to the poor passivation effect in harsh conditions.^[^ ^212^, ^213^ ^]^ To address these issues, different strategies such as designing novel fluorination reaction routes, exploring new fluorination reagents, and developing new types of fluorinated solvents and salts along with current collector modification^[^ ^212^ ^]^ have been widely performed to construct robust fluorinated SEI and improve electrochemical stability. In this regard, the fundamental understanding of fluorinated SEI ought to keep up with practice, including the differences between in situ and ex situ SEI, dynamic evolution of SEI, ion transport mechanism in different electrolyte systems and the decomposition mechanism of solvents and anions. From a practical perspective, it prefers to developing fluorinated interphase engineering to ensure the structural integrity and dendrite‐free morphology of Li metal anode through synergic effects of solvents, anions, additives and other functional components, which is expected to regulate the formation process of SEI by influencing the structure of solvation Li^+^ and form a stable SEI more effectively.

## 6 Perspective and Summary

In this review, we have focused on the functions and applications of fluorinated interphase for advanced rechargeable Li battery, and discussed the synthetic strategies developed for a F‐rich surface film. As for the cathode materials of LIBs, we summarized the current techniques to build metal fluorides nanocoating, based on which the structure–performance relationship was analyzed to highlight the importance of surface control of the high energy cathode materials. The fluoride surface layer showed extraordinary capability to restrain the interfacial side reactions due to its outstanding chemical stability. Meanwhile, it is also important that a delicate control on the metal fluoride surface layer is not only essential, but also is still in emergent need as far as the protection effect is to be optimized especially considering the fact that the surface coating layers themselves are inert in charge transfer. Similarly, as for the Li metal negative side, different methods in form of both ex situ and in situ styles are able to create fluorinated Li metal so as to modulate the deposition behavior of Li on the surface. Although the contribution of the artificial SEIs constructed by the ex situ protocols have been well demonstrated, the formed surface SEIs are unfortunately known to be fragile in nature, and is easy to break down upon long cycles, which is considered to be a reflection of the rigid structure of pure metal fluorides. As a contrast, those artificial SEIs formed from in situ ways through electrolyte design turned out to be more appealing due to the promising characteristics such as multilayer structure, organic–inorganic complex, and high elasticity, which endows the lithium metal with unique capability of mechanical flexibility and self‐healing, thereby providing a powerful tool to ensure stable anode performance through a successful suppression of the plaguing Li dendrite formation.

There still exists enormous opportunity ahead in fluorinated interphase design to deal with formidable challenges related to dendritic initiation, side reactions, and structural degradation. We will list below different research directions that may be helpful for not only deeper understanding of SEI but also further developing fluorinated interphase for lithium secondary battery toward its practical applications.

### 6.1 Solution‐Based Conformal Fluoride Coating for High‐Energy Cathodes

Most of the existing fluoride modifications are based on simple liquid phase mixing strategy, lacking in fine regulation of precipitation process to achieve a controllable growth kinetics and uniform morphology. It is generally observed that the fluoride coatings prepared by this wet mixing‐drying processes display rough or even granular morphology. Consequently, the bare surfaces with limited protective effect in some of the area are exposed to electrolyte and served as sites for harmful reactions. Accordingly, a uniform fluoride coating is necessarily pursued not only for homogenizing surface potential field and alleviating the charge heterogeneity of active particles but also for enhancing structural stability against erosion and internal stress. To this end, it could open up a feasible way through selecting suitable precursors, adjusting reactant concentration/ratio and optimizing precipitation chemical environment. In addition, for those moisture‐sensitive materials, particularly Ni‐rich cathodes, aqueous methods may cause potential harm to their structural integrity due to aggravated Li^+^/H^+^ exchange reactions, demanding refined washing procedure. Therefore, the liquid phase method based on organic solution is an alternative way to avoid the influence of water, which is expected to realize large‐scale production.

### 6.2 Novel Fluorinated SEIs Targeted for Specific Functions

Currently, the reagents to generate fluorinated SEI are limited to few fluorinated solvents and anions, which are not fully meet with the requirements of high‐voltage system. Therefore, it is of great importance that further explorations on electrolyte systems and novel additives be made to broaden the electrochemical stability window, contributing to LMBs with high safety and long lifespan. Furthermore, considering the actual situation of battery operation, it is of necessity to ensure the reliability of SEI as the dynamics of charge/ion transport and the features of surface are varying with external environment. For example, at a low temperature, the fluorinated SEI is prone to be homogenous and crystallized, which are not sufficient to passivate the surface of lithium anode, leading to severe dendrite propagations. While under the circumstance of high temperature, the unstable SEI is vulnerable to the dissolution of components and intensified cointercalation of solvent molecules, resulting in degraded cyclability. An approach to designing novel fluorinated SEI targeted for specific functions is thereby greatly needed for practical applications.

### 6.3 Formation and Evolution Mechanism of SEI

Generally, the formation of SEI involves many complex processes in nanoscale, including solvation/desolvation of Li^+^, adsorption of solvated Li^+^, ionic/molecular distribution between interface electric double layers, intercalation, and decomposition of electrolyte. Meanwhile, in the subsequent electrochemical process, the dissolution of species, the volume change of electrode materials, and the consumption of electrolyte will give rise to the dynamic structural evolution of SEI. However, the corresponding knowledge of these intermediate states is still scarce due to the subtle nature of SEI that suffers from damages made by electron radiation, X‐ray, temperature, and moisture. Therefore, in situ/operando nondestructive characterizations are pivotal in revealing the formation and evolution mechanism of SEI through probing atomic structure and capturing dynamic information, which facilitates further understanding of the relationship between structure and performance. We hope that intensive research progress will be made in SEI from basic recognition to theoretical practice considering that the most important but least understood role of SEI may provide enormous possibilities for future manufacturing of rechargeable batteries with superior properties.

## Conflict of Interest

The authors declare no conflict of interest.
